# Forest degradation drives widespread avian habitat and population declines

**DOI:** 10.1038/s41559-022-01737-8

**Published:** 2022-04-28

**Authors:** Matthew G. Betts, Zhiqiang Yang, Adam S. Hadley, Adam C. Smith, Josée S. Rousseau, Joseph M. Northrup, Joseph J. Nocera, Noel Gorelick, Brian D. Gerber

**Affiliations:** 1 Forest Biodiversity Research Network, Department of Forest Ecosystems and Society, Corvallis, OR USA; 2 United States Forest Service, Rocky Mountain Research Station, Ogden, UT USA; 3 New Brunswick Department of Natural Resources and Energy Development, Fredericton, New Brunswick Canada; 4 National Wildlife Research Centre, Environment and Climate Change Canada, Ottawa, Ontario Canada; 5 Cornell Lab of Ornithology, Ithaca, NY USA; 6 Ontario Ministry of Natural Resources and Forestry, Peterborough, Ontario Canada; 7 Faculty of Forestry and Environmental Management, University of New Brunswick, Fredericton, New Brunswick Canada; 8 Google Earth Engine, Zurich, Switzerland; 9 Department of Natural Resources Science, University of Rhode Island, Kingston, RI USA

**Keywords:** Conservation biology, Conservation biology

## Abstract

In many regions of the world, forest management has reduced old forest and simplified forest structure and composition. We hypothesized that such forest degradation has resulted in long-term habitat loss for forest-associated bird species of eastern Canada (130,017 km^2^) which, in turn, has caused bird-population declines. Despite little change in overall forest cover, we found substantial reductions in old forest as a result of frequent clear-cutting and a broad-scale transformation to intensified forestry. Back-cast species distribution models revealed that breeding habitat loss occurred for 66% of the 54 most common species from 1985 to 2020 and was strongly associated with reduction in old age classes. Using a long-term, independent dataset, we found that habitat amount predicted population size for 94% of species, and habitat loss was associated with population declines for old-forest species. Forest degradation may therefore be a primary cause of biodiversity decline in managed forest landscapes.

## Abstract (web summary)

A study in eastern Canada finds that forest-management strategies that lead to simplified forest structure and composition have resulted in loss of breeding habitat and associated population losses for many bird species.

## Main

Most conservation policies have focused on reducing deforestation (that is, permanent conversion to another land-cover type), and this approach remains fundamental to many conservation strategies. Effects of forest loss on global biodiversity are well known, directly measured^1^ and often used as estimates of biodiversity decline^2^. Forest degradation is also expected to be a key driver of biodiversity decline and is a component of broad-scale biodiversity agreements (for example, Aichi Biodiversity Targets in the Convention on Biological Diversity, REDD + [Reducing Emissions from Deforestation and Forest Degradation]). However, forest degradation has been much more challenging to measure, and there have been few attempts to quantify its effects on species’ population trends across entire regions^3,4^.

**Fig. 1:**
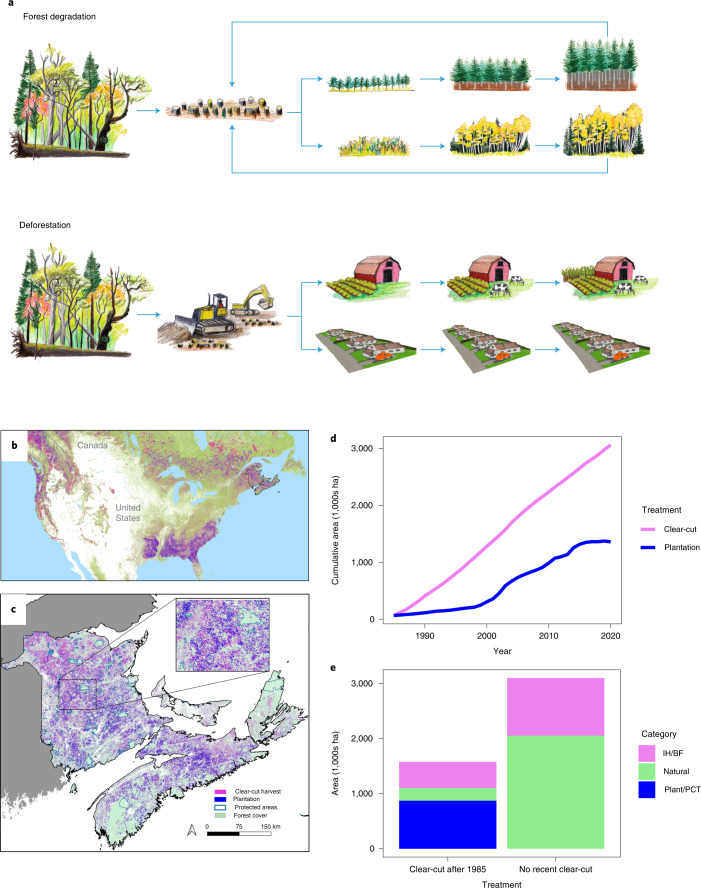
Forest management as a potential cause of forest degradation. **a**, Conceptual diagram showing the contrast between forest degradation and deforestation^24^; in eastern Canada, degradation generally results from clear-cutting of original forest followed by either tree plantations or natural regeneration of pioneer tree species. Age–class truncation takes place when regenerated forests are clear-cut before developing the composition and structure of the original forest (reverse arrows). Alternatively, deforestation occurs when forest is replaced by another land-cover type (for example, urban or agricultural areas). Drawing credit: Deirdre Hyde. **b**, The study area in context of other regions of North America that have similar rapid rates of forest loss (pink) then gain (purple), which is probably a signal of commercial forest harvest followed by rapid regeneration (data from: www.globalforestwatch.org). **c**, Cumulative clear-cut disturbance across the Maritime provinces of eastern Canada from 1985 to 2020 (pink) along with the area that has been converted to plantations (blue). **d**, Cumulative area clear-cut and planted across the study area over the same time period. Methods for mapping plantations and disturbance are given in the Supplementary Methods. **e**, The area of forest that has been clear-cut since 1985 (left bar) for public land and private wood lots for a subset of the study area (New Brunswick; 72,908 km^2^) and forests that have not been clear-cut since that date (right bar). Most forest cut since 1985 has been planted or pre-commercially thinned (PCT) to favour conifer species (blue bar) or has regenerated as shade-intolerant hardwood (IH) or balsam fir (*Abies balsamea*, BF; pink bar). In contrast, forest that has not been recently clear-cut is comprised of shade-tolerant tree species (green bar). Intolerant hardwood/balsam fir stands in areas not recently harvested probably originated from disturbances before 1985. Data in **e** were derived from the New Brunswick Forest Inventory (2010) and do not include changes over the past decade.

From a biodiversity standpoint, forest degradation is defined as the reduction or loss of biological complexity in forests^5^. Forest management alters forest complexity most commonly in two important ways; first, due to harvesting, managed forests tend to be younger than those under a natural disturbance regime^6^ with potential implications for species associated with mature or old-growth forests^7^. Second, because intensive silviculture such as tree planting and thinning tend to yield more wood per area, managers increasingly convert native forests to plantations^8^. Unlike most natural forests, plantations tend to be comprised of only one or two tree species, and thinning is used to shift composition towards merchantable species, thereby simplifying forest composition (Fig. 1). Plantation area is expected to rise as plantations are increasingly considered ‘natural climate solutions’^9^. Such changes in age–class structure and forest composition may occur without any overall loss in forest cover and have thus been largely ignored^4^. Nevertheless, quantifying forest degradation is of critical importance to understanding biodiversity responses in regions where timber harvest and regrowth predominate (for example, Canada, western United States, Scandinavia, Russia)^10^.

The importance of quantifying forest degradation effects is particularly critical considering recent findings by the Intergovernmental Panel on Biodiversity and Ecosystem Services that the planet is facing a biodiversity crisis^11^. Causes of population declines remain poorly understood for many species, including birds, which have experienced widespread but cryptic population declines over the past three decades^12^.

The hypothesis that breeding habitat loss and resultant population declines are driven by forest degradation remains largely untested. This is likely for two methodological reasons. First, changes in forest composition and age–class structure due to forest management are more challenging to detect than deforestation^13^. Managed forests tend to be highly dynamic ‘shifting mosaics’ in space and time, with new harvests occurring regularly and then regenerating along various successional trajectories^14^. To properly characterize the effects of these changes on animal populations, broad-scale spatial forest inventories would be necessary at fine temporal resolutions relevant to particular taxa. To our knowledge, such data are rarely, if ever, available.

**Fig. 2:**
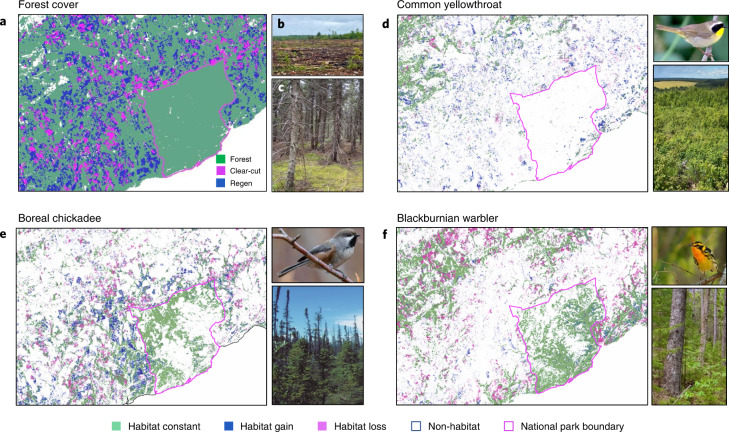
The importance of a species-centred approach to detecting effects of forest degradation. **a**–**f**, Maps showing forest cover (green in **a**), recent clear-cuts (pink in **a**; **b**) and >two-year-old clear-cuts planted, thinned or regenerating (Regen) naturally (blue in **a**; **c**) in relation to SDM-predicted habitat and habitat changes (1985–2020) for: common yellowthroat (**d**), which is associated with young deciduous forest (net regional habitat gain = +8.3%), boreal chickadee (**e**), associated with old conifer forest (net regional habitat loss = −19.0%) and Blackburnian warbler (**f**), associated with old mixed coniferous/deciduous forest (net regional habitat loss = −33%); see adjacent photos of species-associated forest types. Due to habitat specialization (adaptation to particular forest types and age classes), each species is distributed uniquely across forest landscapes and therefore is differentially affected by clear-cuts and regeneration (**a**). Using coarse definitions of forest change (for example, forest loss or cover) will not effectively quantify species-specific habitat changes over time. SDMs based on Landsat variables enable quantification of annual habitat amounts and the direct effects of spatially congruent forest degradation (for example, changes in structure and composition initiated by clear-cut disturbance) on habitat for each species. Thresholds for quantification of habitat versus non-habitat are provided in Supplementary Table 1. The legend for habitat maps is provided below the figure. Photo credits: boreal chickadee, Iris Kilpatrick; all other photos, M.G.B.

Second, it is well known that species have different habitat requirements, which often do not correspond to coarse, human-defined land-cover categories (for example, forest, urban, agriculture)^15^ or even coarse forest-inventory categories (for example, ‘young’, ‘old’, ‘deciduous’, ‘coniferous’ and so on; Fig. 2). Species show various degrees of association across gradients in forest age and compositions^16^. Indeed, only the most generalized forest species can be found across all forest types and age classes.

Here we navigated these previous obstacles to quantifying forest degradation effects on species’ habitat and populations by applying a ‘species-centred approach’^15^. We used Landsat Thematic Mapper (TM) reflectance bands as predictor variables in species distribution models (SDMs) to quantify species-specific habitat for 54 forest-dependent bird species in the Acadian Forest of eastern Canada (130,017 km^2^). Here we define habitat as a species-specific concept that reflects the conditions necessary to enable potential or actual occupancy of a given organism^17^. Landsat bands have been used extensively to detect land-cover change—particularly forest composition, disturbance and regrowth^13^—and have been successfully used to model forest bird habitat directly^18^. Unlike spatial forest-inventory data, Landsat data are available annually since 1985; this enabled us to back cast SDM predictions to quantify habitat changes for each species over 35 years (1985–2020).

Under the hypothesis that forest degradation is driving habitat loss and population declines, we predict that we should see (1) little net change in total forest area (due to the rates of forest regeneration matching forest harvest), (2) loss of old forest due to high harvest rates (short harvest rotation intervals), (3) reductions in breeding habitat across forest-associated species, particularly those associated with mature native forest, which is under pressure from timber harvest, (4) correlations between habitat amount and bird abundance over the 1985–2019 period as quantified in an independent dataset, the North American Breeding Bird Survey^19^ (BBS) and (5) direct negative effects of habitat loss on inter-annual bird-abundance changes.

## Results

**Fig. 3:**
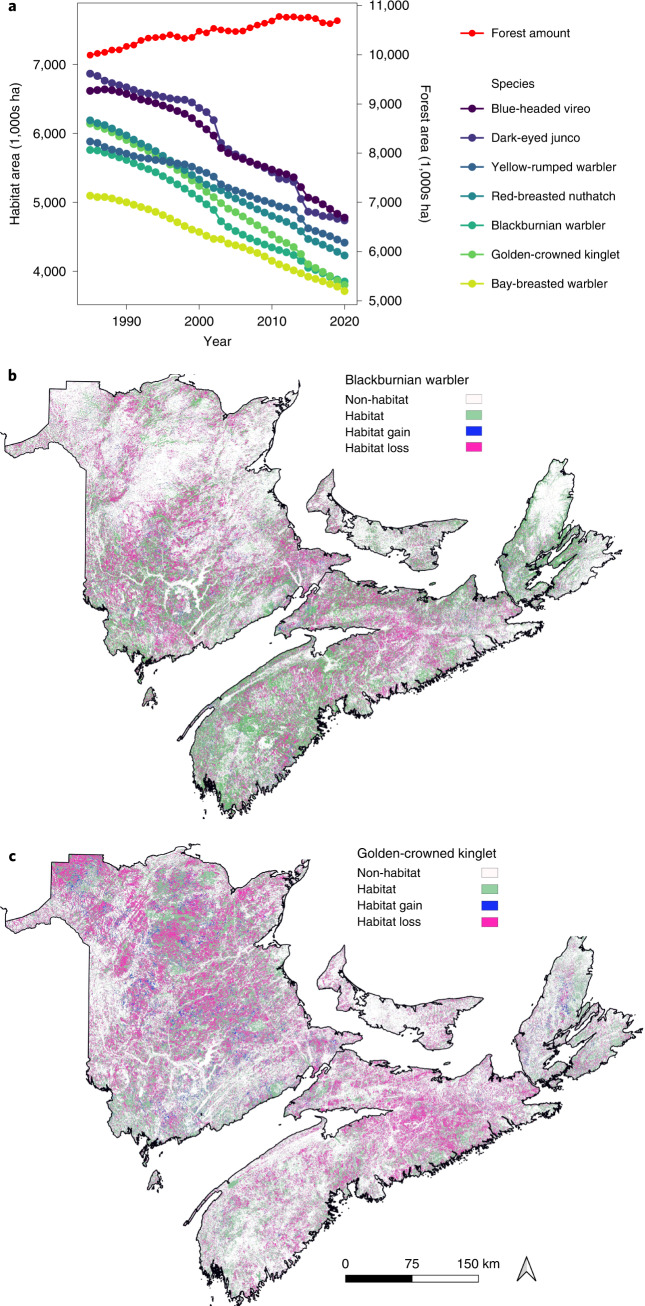
Forest degradation rather than loss drives habitat declines in old forest-associated bird species. **a**, Habitat trends (1985–2020) for the seven bird species exhibiting the greatest population declines according to SDMs; all of these species are old forest associated. During the same time interval, total forest cover did not decline (red line, right axis), indicating that habitat loss is a function of forest degradation rather than loss. **b**,**c**, Predicted habitat loss (pink) and gain (blue) between 1985 and 2020 for two example species: Blackburnian warbler (33% habitat loss; **b**) and golden-crowned kinglet (38% habitat loss; **c**). Habitat loss was quantified using SDMs with Landsat data as independent variables strongly predicted population trends for forest bird species.

**Extended Data Fig. 1:**
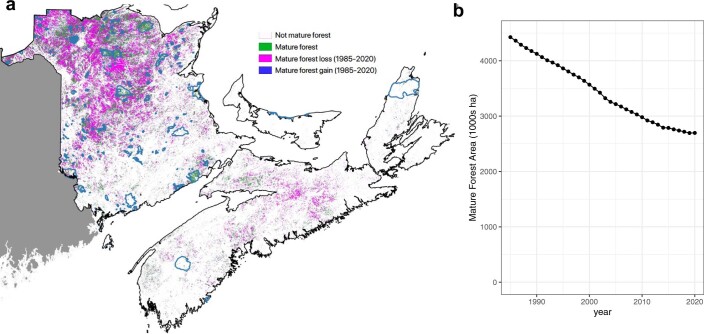
Map showing change in mature forest across the Maritime provinces of eastern Canada. National parks and other protected areas (for New Brunswick only) are outlined in blue. Panel b: Overall, mature forest exhibited a net decline of 39% from 1985–2020. This decline is primarily due to clearcut harvesting (see Fig. 1a) and insufficient recruitment of forest into older age-class categories as a result of short harvest rotations. See Supplementary Methods (‘Old Forest Types’) for details on how old forest loss was quantified. Training data were only available for New Brunswick (the western part of the study area) so extrapolation was necessary for estimates of mature forest in Nova Scotia and Prince Edward Island (the eastern part of the study area).

The Acadian Forest of eastern Canada has shown a pervasive signal of forest degradation since 1985 (Fig. 1). Since 1985, >3 million ha have been clear-cut (Fig. 1d), with most of this area now occupied by either tree plantations and thinnings (Fig. 1c–e), which are dominated by single tree species^20^, or a mix of early successional tree species (Fig. 1a,d,e). Despite some ingrowth due to succession, old forest has declined by 39% during the period observed (Extended Data Fig. 1a,b; Supplementary Methods). The pattern of extensive harvest of old forest, followed by rapid regeneration of young forest appears to be common across many forest regions of North America (for example, central Canada, southeastern United States, western United States; Fig. 1b) (ref. ^10^) and can be considered ‘forest degradation’ in that these practices simplify forest structure, reduce tree species diversity and truncate old-forest age classes^6^. During the same 35-year time period, forest cover remained relatively stable, increasing by a net 6.5% (Fig. 3a, red line)^21^.

**Extended Data Fig. 2:**
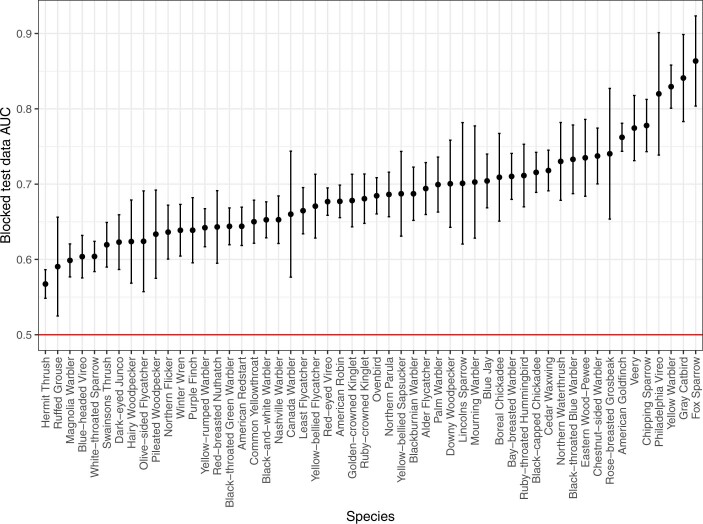
Area Under the Receiver Operating Characteristic Curve (AUC) – a measure of model prediction success ranging from 0-1 (perfect predictions) – for presence-only species distribution models with 54 forest bird species of the Maritime Provinces. AUCs were calculated using 50% (N = 66,136) of avian point count locations held-out from test data in 15 km^2^ blocks to ensure spatial independence.

Overall, SDMs using Landsat reflectance bands as predictors performed well for most forest bird species when tested on 50% spatially discrete hold-out data (Extended Data Fig. 2; $\bar x$ area under the curve (AUC) = 0.73 [range: 0.60–0.90]). SDMs therefore provided reliable estimates of habitat suitability and distribution for most of the 54 species. Species with lower model-prediction success tended to be associated with fine-scale forest structure (for example, individual tall trees, standing and fallen dead wood) which are poorly captured by satellite imagery.

**Fig. 4:**
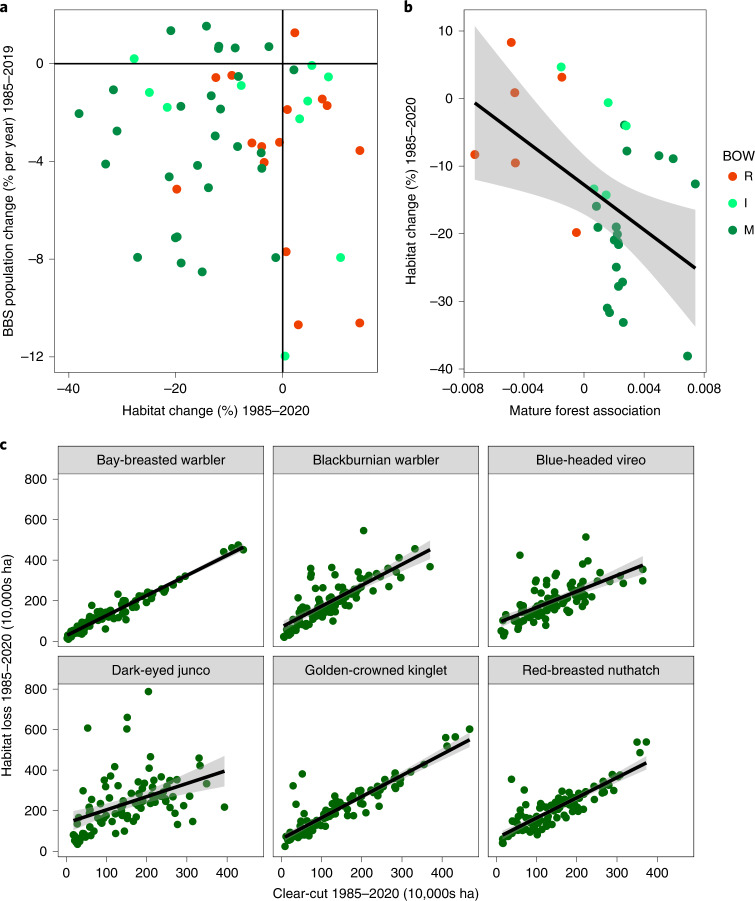
Evidence for the effect of forest degradation on mature-forest bird species. **a**, The relationship between habitat change, estimated from SDMs and independently derived population change estimates from the BBS for the Acadian forest. Bird species of mature (old) forests (M; dark green dots) exhibit the greatest habitat loss; this is generally reflected in strongly negative population trends. Bird species associated with regenerating forest (R; red dots) tend to have stable or increasing habitat but still show BBS population declines. **b**, The relationship between quantitatively derived estimates of mature-forest association and habitat change from 1985 to 2020. Mature forest-associated species tend to be losing the most habitat in relation to immature- (I; light-green dots) and regeneration-associated species. Successional stage categorizations (R, I, M) are from Birds of the World (BOW). The regression line was fit using a hierarchical Bayesian model (Supplementary Methods) and grey shading in **b** shows 95% credible intervals. Only a subset of species is shown in **b** (those with quantitative data for mature-forest associations; Supplementary Methods). **c**, The relationship between area clear-cut occurring from 1985 to 2020 in each species’ habitat within a 200 m-diameter buffer surrounding BBS routes (*N* = 90) and habitat loss (1985–2020) at the same scale for six mature forest-associated species. Black lines are regression lines and grey bands are 95% confidence intervals (regression estimates in Supplementary Table 3). As expected, clear-cutting is strongly associated with habitat loss, which indicates that ingrowth of new habitat is rarely compensated for by habitat loss (a signature of forest degradation via old age–class truncation).

**Extended Data Fig. 3:**
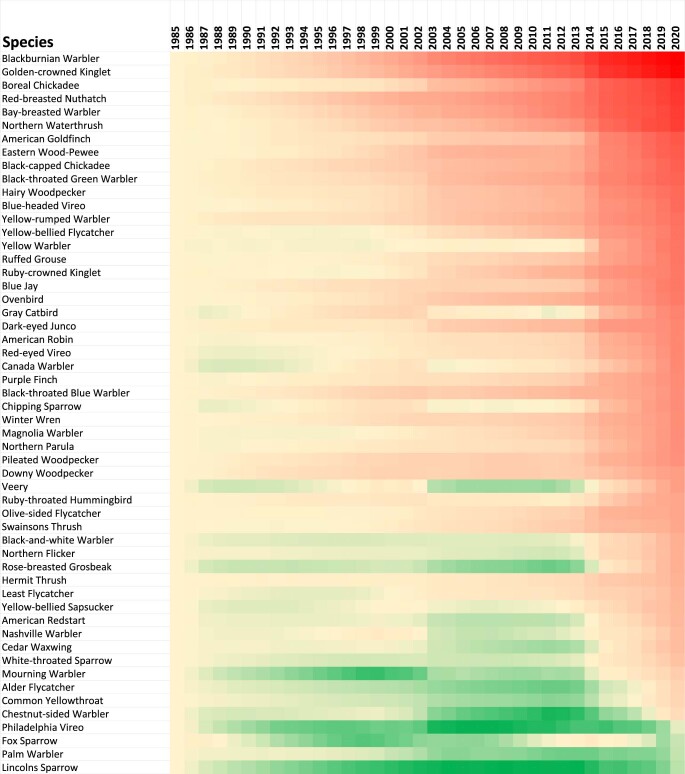
Habitat change (1985–2020) for 54 species of forest birds according to back-cast species distribution models. Transitions from green, through yellow, to red across cells indicate annual habitat loss. Sixty-six percent of species show net habitat loss over the full time period, and 93% lost habitat over the past 10 years.

**Extended Data Fig. 4:**
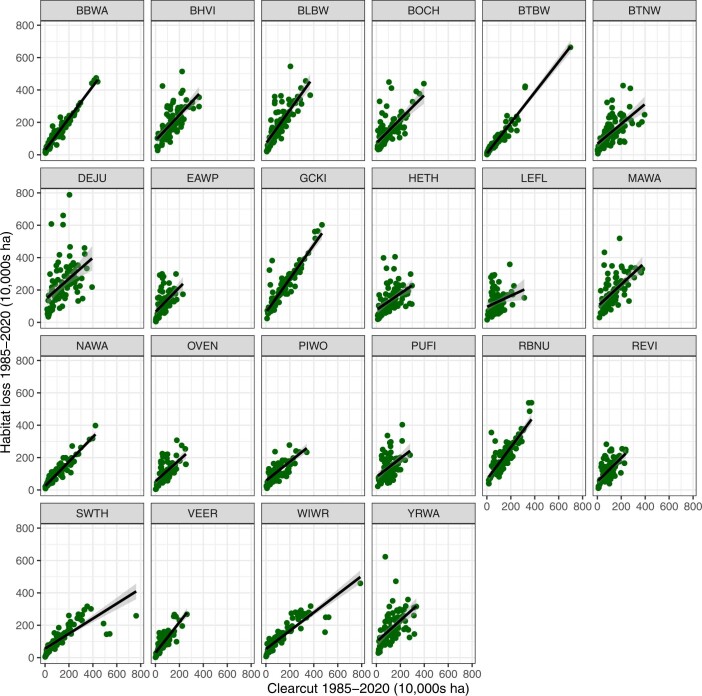
Relationship between area clearcut occurring from 1985–2020 in each species’ habitat in a 200 m buffer surrounding Breeding Bird Survey Routes (N = 90) and habitat loss (1985–2020) at the same scale for each of 23 mature-forest associated species (species codes provided in Supplementary Table 5). Black lines are regression lines and gray bands are 95% confidence intervals. This relationship would have been obscured if the rate of habitat gain (regrowth) compensated for habitat loss (due to clearcutting mature forest). However, in this system, clearcutting is removing habitat without compensatory replacement.

**Extended Data Fig. 5:**
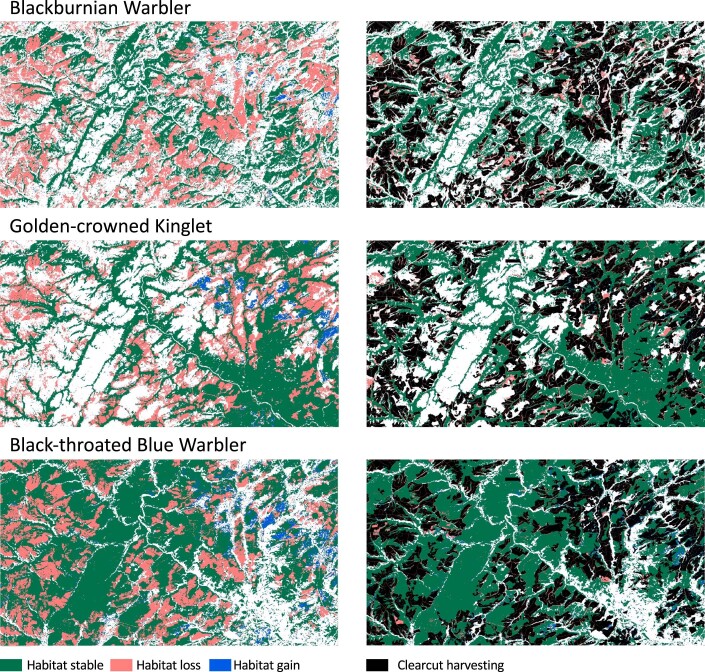
Spatial association between clearcutting and habitat change for three mature forest species. The first column of panels shows species-specific habitat that has remained stable since 1985 in green with loss of habitat in pink and habitat gains in blue. The second column of panels shows the footprint of clearcut harvesting (black) within the same landscape. Remaining areas in pink in the right-hand column are locations where habitat has been lost due to a different cause than clearcutting (for example, land-use change). There is high congruence between clearcutting and areas where habitat was lost. Clearcutting data shown are from the New Brunswick provincial inventory.

We back cast SDMs to quantify habitat change for all 54 forest bird species from 1985 to 2020. Habitat declines occurred for 66% of species during 1985–2020; 93% of species exhibited habitat reductions over the past decade (Fig. 3 and Extended Data Fig. 3). Species showing the greatest decreases in habitat were golden-crowned kinglet (*Regulus satrapa*; −38%) and Blackburnian warbler (*Setophaga fusca*; −33%; Supplementary Video 1) with seven species showing habitat declines >25% (Fig. 3). Most species with strongly declining habitat are associated with old forests^22^ (Fig. 4a,b), which is consistent with forest degradation due to harvesting of old forest. Indeed, clear-cut harvest alone was strongly associated with habitat declines for all old forest-associated species (Fig. 4c and Extended Data Figs. 4 and 5). Forest succession into old age classes was apparently insufficient to compensate for this rate of loss. Fifteen species exhibited habitat increases, but most (14 out of 15) of these tend to be associated with young or immature forests (Fig. 4a,b).

**Extended Data Fig. 6:**
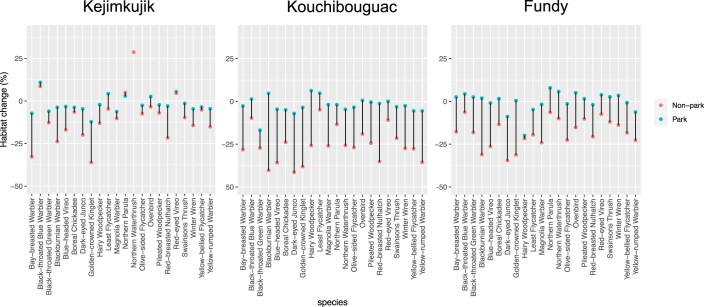
Habitat change for 22 species of old-forest associated birds both inside (green) and outside (within a 50 km buffer) of three large terrestrial national parks in the Maritime provinces. Under the hypothesis that timber harvest and forest management are the primary drivers of habitat decline, loss should be predominantly outside of reserves, where harvesting is not permitted. Although for some species, minor habitat loss occurs inside parks (likely due to natural shifts in forest composition), habitat loss is much higher beyond park boundaries.

**Extended Data Fig. 7:**
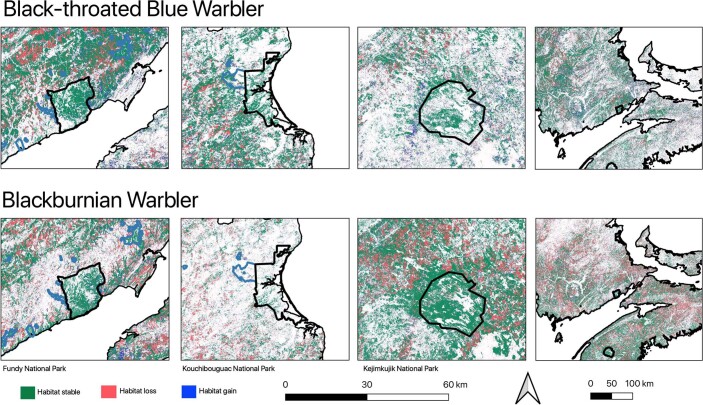
Habitat distribution and change maps for two examples of mature-forest-associated species within and outside three national parks in eastern Canada (Fundy, Kouchibouguac, Kejimkujik National Parks) and the core area of the study region. Note that habitat loss (red) is common in landscapes surrounding parks, but largely absent within, indicating that the habitat loss we quantified is due to timber harvest, not climate-induced changes in Landsat reflectance, or natural disturbance. White areas indicate non-habitat.

Several lines of evidence support forest management as the primary driver of forest degradation rather than alternative mechanisms (for example, climate-mediated forest decline, natural disturbance, permanent deforestation). First, our SDMs did not include climate data so the reflectance changes from satellite imagery used in our SDMs were predominantly due to forest compositional changes. Although climate (for example, inter-annual differences in precipitation) can cause subtle differences in reflectance (leaf colour) over time, most changes in the magnitude of reflectance are due to changes in forest composition or cover rather than effects of climate^23^ (Supplementary Figs. 1 and 2). Indeed, if the observed habitat declines were due to climate effects or natural disturbance, we would expect to see parallel habitat declines in protected areas, which we did not (Extended Data Figs. 6 and 7). Second, species exhibiting the greatest declines in habitat are those most strongly associated with old forest (Fig. 4a,b), which is the primary target of timber harvest. Indeed, the amount of area clear-cut was strongly associated with habitat loss for old forest-associated bird species (Fig. 4c and Extended Data Figs. 4 and 5). Third, deforestation (defined as permanent conversion to another land-cover type)^24^ was not a primary driver of habitat loss in our region; deforestation contributed <2% of total habitat loss for all 54 species (Supplementary Information and Supplementary Table 2). We acknowledge that due to the complex nature of changes in forest structure and composition through forest management, our evidence for forest-management effects on bird habitat is necessarily indirect. However, given the apparent minimal effects of climate and deforestation on habitat change, forestry-driven degradation is the most parsimonious remaining explanation for substantial habitat declines.

Next, we tested the hypothesis that habitat loss was positively correlated with bird-population declines using BBS data for the Maritime provinces (Methods). We used SDMs to quantify habitat change (1985–2019) in landscapes surrounding BBS routes (*N* = 90; Supplementary Methods). We then used Bayesian hierarchical models^19,25^ in a space-for-time approach to test whether the SDM-predicted habitat amount in each year of the time series was associated with population size for each species along each route. Importantly, BBS data are entirely independent of our SDMs, so this test also represents a strong validation of our SDM-derived habitat models. Second, using an additional model parameter, we tested whether annual change in SDM-modelled habitat changes (increases or decreases) along routes in each year could predict annual bird-abundance changes.

**Fig. 5:**
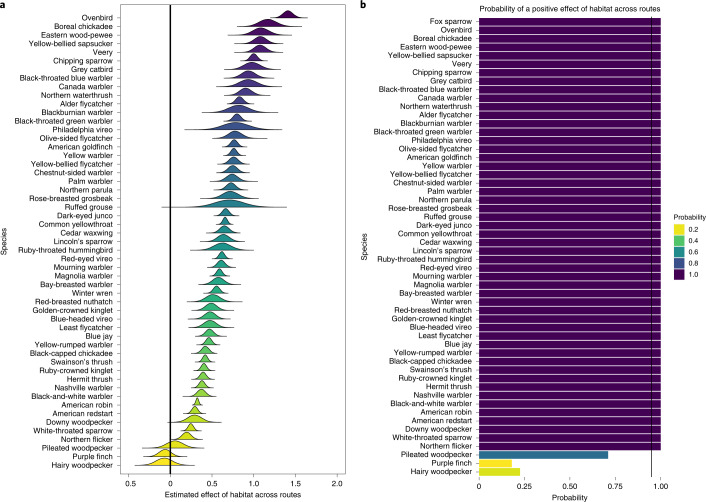
Positive effects of habitat amount on bird-population abundance. **a**, Posterior distributions for the effects of SDM-derived habitat amount across routes (*x* axis) on bird abundance, using BBS data. The vertical black line at zero reflects no positive or negative population trend. Abundance of most species was positively influenced by habitat, which supports the hypothesis that bird populations are strongly linked to breeding habitat amount. **b**, The posterior probability that habitat had an effect on population size for 54 forest bird species. The vertical black line indicates 95% posterior probability of an effect.

Bayesian models revealed a strong effect of habitat amount on BBS bird abundance for all but three species (Fig. 5). Abundance of all but three species tracked annual habitat amount with 95% posterior distributions that did not include zero (vertical line in Fig. 5a; posterior probability, Fig. 5b). The effect of habitat was substantial and probably biologically meaningful for most species, with abundance decreasing a median of 7.99 times from landscapes with the highest to lowest habitat amounts (Supplementary Table 4).

**Extended Data Fig. 8:**
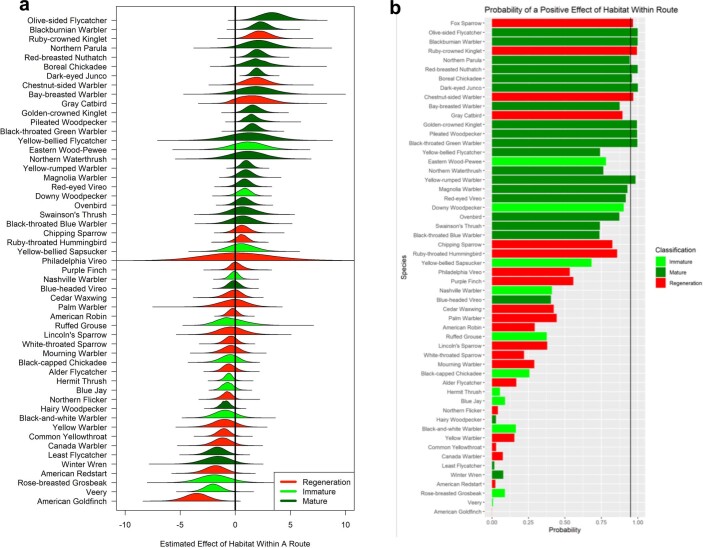
Effects of habitat change on changes in bird population abundance. Panel **a** shows Bayesian posterior distributions for the effects of SDMmodeled habitat change (x-axis) in each year on bird abundance in the corresponding year (parameter µ_β_ in Equation 3), using Breeding Bird Survey data. Population changes for thirteen species were strongly positively influenced by habitat changes on abundance; most of these species (10/13) are associated with old forest (dark green) which supports the hypothesis that forest degradation-driven declines in habitat amount are affecting population changes in these species. Panel b shows the posterior probability that habitat change had an effect on population change for 54 forest bird species. Fox Sparrow not shown in a due to large positive effect size.

We also found that annual changes in habitat along BBS routes were associated with bird-abundance changes (Extended Data Fig. 8); in other words, habitat loss in one year resulted in abundance declines along routes in the same year. For thirteen species, the Bayesian estimate for the effect of habitat loss on population decline had posterior probabilities >0.95, and 20 species had posterior probabilities >0.8. Importantly, most of the species showing an effect of habitat loss along routes on changes in population decline have lost substantial habitat over the time period and are associated with old forest (for example, Blackburnian warbler, northern parula [*Setophaga americana*], red-breasted nuthatch [*Sitta canadensis*], boreal chickadee [*Poecile hudsonicus*], dark-eyed junco [*Junco hyemalis*]; Extended Data Fig. 8), which would be expected with the harvest of old forest—a component of forest degradation. It is important to note that this test is highly challenging because many factors can drive annual fluctuations in bird abundance (for example, weather, phenology, conditions during migration or on the wintering grounds). Also, in any given year, habitat change along BBS routes can be quite small for some species; this low inter-annual variation in a predictor variable can preclude high statistical power to detect effects.

We estimated the net number of breeding individuals that have probably disappeared due to habitat loss from 1985 to 2020 using published accounts of territory sizes for each species^22^ (Supplementary Table 5). This calculation assumes that available habitat is consistently occupied, which is supported by strong associations between habitat amount along BBS routes and bird abundance over the long term. Across all species, back-cast SDMs indicate that a net 28,215,247 ha (282,153 km^2^) of habitat has been lost, equating to a loss of between 16,779,704 and 52,243,938 breeding pairs (33,559,408–104,487,876 individuals; Supplementary Methods and Supplementary Table 5). One might expect that forest degradation, rather than resulting in broad-scale declines across species, is simply causing species turnover from old forest-associated bird species to young-forest associates. However, it is important to note that we quantified *net* bird decline from an unbiased list of the 54 most common forest bird species in eastern Canada. This list included both early and late successional species. Such net bird declines could be due to the fact that (1) even some early seral species are losing habitat (probably due to conversion from diverse early successional forest to species-poor plantations and thinnings)^26^ and (2) in this region, more species occupy older forests than regenerating forests^27^.

**Fig. 6:**
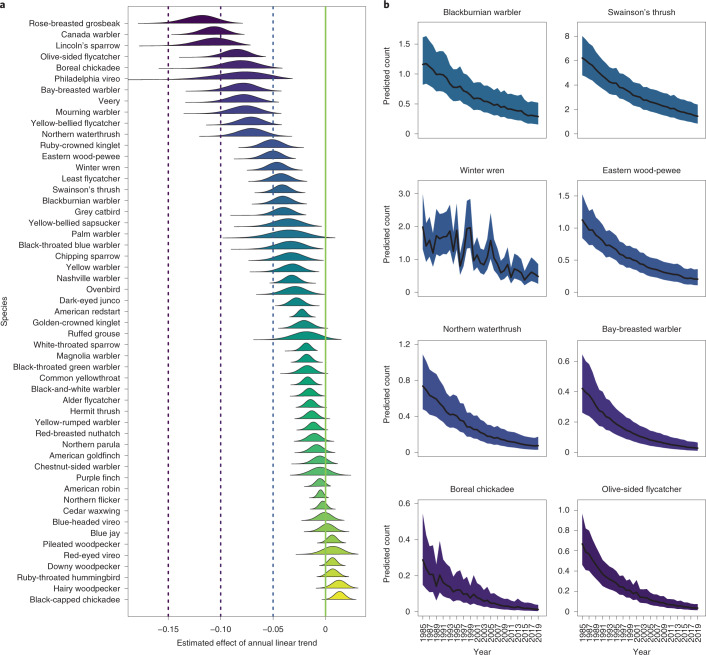
Population trends for forest-associated birds in eastern Canada. **a**, Population trend parameter estimates and posterior distributions for 54 species of forest birds derived from Bayesian models. Seventy-two percent of species that are sufficiently common to model experienced population declines from 1985 to 2019. Colour key is provided in Fig. 5. The vertical green line indicates a population trend of zero. Dashed vertical lines coincide with trends of −15% (−0.15), −10% (−0.10) and −5% (−0.05) annual population trends. **b**, Predicted linear population trends for 1985–2019 (regression lines are mean trends derived from Bayesian Poisson models, Supplementary Methods) including annual variation estimated from BBS data. Shaded purple areas reflect 95% credible intervals and reflect the magnitude of species population declines shown in **a**. Populations of these eight old forest-associated species have declined 60–90% over the period observed.

We also quantified overall population trends for 54 species of forest birds using data from the BBS (Fig. 6). These estimates give the total magnitude of population changes which include, but are not limited to, habitat loss or gain effects. Thirty-nine of the 54 species examined (72%) are in population decline (defined as having 95% credible intervals that do not bound zero). The magnitude of the declines for 15 forest bird species is severe (>5% per year). It is notable that most species exhibiting both habitat loss *and* population declines are old-forest associates (Fig. 4a; bottom left quadrant, dark green dots), with old-forest species exhibiting the greatest habitat losses (Fig. 4b and Supplementary Methods; hierarchical regression, $\hat \beta$ = −16.66 [6.32 SE]).

**Extended Data Fig. 9:**
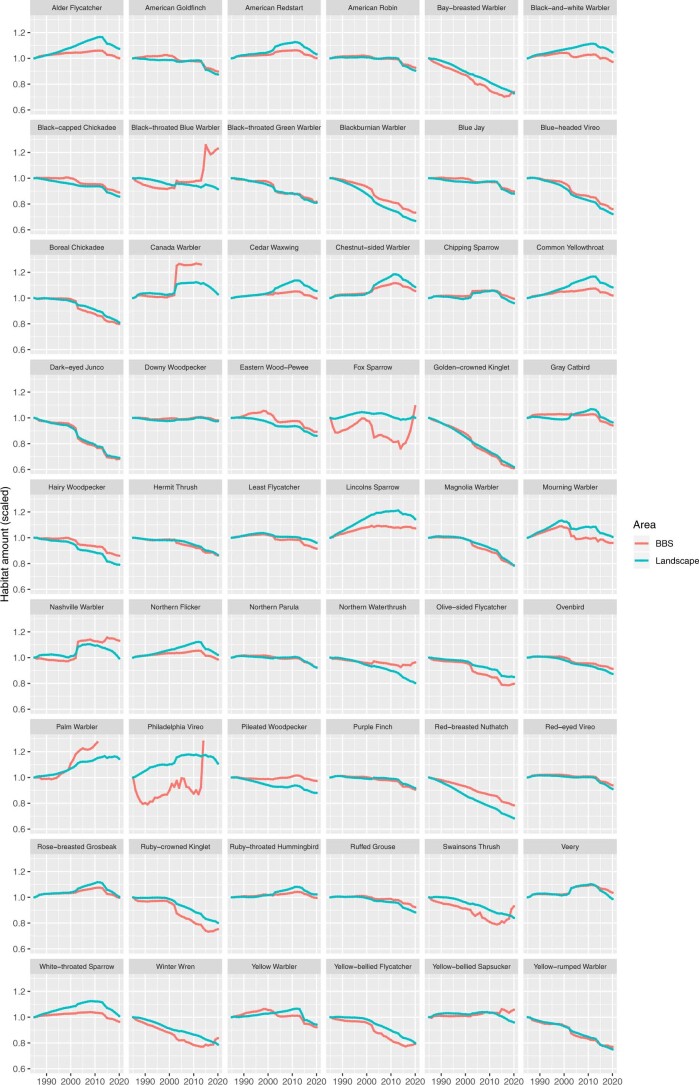
Habitat trends within 100 m of BBS routes (red lines) versus the entire Maritimes region (green lines) for 54 species of forest birds. Habitat trends along BBS routes tend to reflect changes in the region except for a few species (for example, Blackthroated Blue Warbler). Habitat amounts for BBS routes and the Maritimes region were normalized to 1 for the starting year (1985).

BBS declines are not restricted to old-forest species; several species in rapid population decline are early seral species (for example, Lincoln’s sparrow [*Melospiza lincolnii*], mourning warbler [*Geothlypis philadelphia*]; Fig. 4a, bottom right quadrant). Despite the fact that these species have gained habitat over 35 years, their populations continue to decline. Only three species (black-capped chickadee [*Poecile atricapillus*], hairy woodpecker [*Leuconotopicus villosus*] and ruby-throated hummingbird [*Archilochus colubris*]) are increasing in abundance. Populations of these species increased despite evidence of habitat decline (Fig. 4a, top left quadrant)—perhaps because each benefit from anthropogenic habitats and supplemental food. Importantly, habitat changes from 1985 to 2019 along BBS routes were representative of changes at the scale of the entire region for most species (Extended Data Fig. 9), so BBS population trends are highly likely to reflect population trends at the regional scale. This contrasts to the 1965–1985 period when mature-forest loss along routes was slower than in the broader region^28^.

We also modelled BBS population trends over the past ten years, as this is the period of importance for informing listing decisions under the Committee on the Status of Endangered Wildlife in Canada (COSEWIC). Nine species have exhibited population declines >30% over ten years (Supplementary Fig. 3), which meets the criterion for consideration as ‘threatened’ under COSEWIC Criterion A (ref. ^29^).

## Discussion

Overall, our results indicate that forest degradation has led to habitat declines for the majority of forest bird species with negative consequences for bird populations, particularly species associated with older forest. Forest changes include conversion from mixed-species forests to single-species conifer-dominated plantations or thinnings (Fig. 1c,d,e) and clear-cutting old forests without equivalent regrowth into old age classes (Fig. 1c,d and Extended Data Fig. 1). Notably, over the same time period, forest cover changed very little (Fig. 3a), and harvest practices in this region are considered sustainable from a wood-production standpoint^30^.

The habitat changes we observed were strongly associated with population size for most forest bird species in our study and appear to be driving population declines (Fig. 5 and Extended Data Fig. 8) in several species, including those associated with old forest (for example, bay-breasted warbler [*Setophaga castanea*], Blackburnian warbler, boreal chickadee, winter wren [*Troglodytes hiemalis*]). Populations of four old forest-associated species are declining at rates >30% over the past ten years (Supplementary Fig. 3), which is a rate consistent with the ‘threatened’ COSEWIC status. We recommend extending the approach we used here to model habitat and habitat change across eastern North America, which encompasses most of the ranges of species in this study. This analysis could be of great importance to future listing decisions.

The strong link between habitat in landscapes surrounding BBS routes and bird-population size indicates that SDMs are highly predictive of bird populations. This finding indicates that breeding habitat loss due to forest degradation is probably a primary cause of observed widespread population declines in birds^12^.

For several species, rates of population decline seemed to outpace rates of habitat decline (compare *x* and *y* axes in Fig. 4a). For instance, Blackburnian warbler populations have experienced an ~70% decline over 35 years (4.5% per year; Fig. 6b), but only 33% of habitat has been lost. One explanation for this apparent mismatch is that populations show particularly strong declines at low^31^ or moderate^32^ habitat amounts (the ‘extinction threshold’ hypothesis)^31^. However, the mismatch between population versus habitat declines could signal that additional, non-habitat-related factors are compounding declines^25^. In support of this idea, several species that that have relatively stable habitats are nevertheless in strong population decline according to the BBS (for example, Canada warbler, Lincoln’s sparrow, Philadelphia vireo [*Vireo philadelphicus*]). Our results do not preclude the effects of wintering ground habitat loss^33^, climate change^34^, mortality on migration^35^ or contaminants^36^. Population declines in species associated with regenerating forest are particularly cryptic because habitat amount for these species tends to be increasing. One hypothesis is that populations of some species that prefer early seral stages, despite having potentially more habitat, are declining due to climate change over the past three-and-a-half decades (~1 °C increase over 30 years^37^) Given that such stand types are probably warmer due to more open and/or shallow canopies^38^, any increases in ambient temperatures are likely to be more severe in plantations and naturally regenerating forests than in mature forests, which would exert physiological stresses and potentially have population consequences on birds. This effect could be magnified by the fact that several early seral species are more associated with young coniferous forest, which is typically found further to the north in boreal forests^20^.

More subtle mechanisms for habitat loss due to forest degradation reported in this study probably would have remained undetectable without a species-specific habitat modelling approach. Given that no two species associate with identical habitats^15^, our model enabled us to quantify habitat on a species-specific basis using SDMs and then track habitat change over multiple decades (since 1985, the origin of Landsat). If we had used generic, human-defined cover types (for example, ‘forest’ or ‘mature forest’) as predictor variables, species-specific patterns in habitat change would have been obscured. Similar approaches could be applied in other regions and for other taxa if species spatial distribution data are available.

It is well established that large-scale intensive forest-management practices in this region have resulted in substantial increases in single-species tree plantations (Fig. 1c–e) (ref. ^20^). In areas that have not been planted, ingrowth of shade-intolerant hardwoods and balsam fir (*Abies balsamea*) predominate; these replace original shade-tolerant deciduous and coniferous species (Fig. 1a) and are unlikely to be succeeded by shade-tolerant species given current short harvest rotations. We predict that similar effects of forest change could be prevalent in other temperate forests globally that are heavily managed for timber production (for example, southeastern United States, Pacific Northwest United States, Chile, Scandinavia). These regions show little net loss of forest cover but high rates of forest reductions and regrowth (for example, Fig. 1b) (ref. ^10^), which is symptomatic of intensive forest management with the potential for forest degradation.

Overall, our results point to broad-scale declines in forest birds of the Acadian forest of eastern Canada. For most species we assessed, abundance is strongly associated with habitat amount, which is affected strongly by forest degradation rather than forest loss. We expect that similar consequences for biodiversity may hold in other intensively managed forests of the world. This mechanism for bird-population declines would have been invisible using coarse, human-defined categories of ‘habitat’ (that is, forest cover).

If maintaining non-declining populations of forest birds is the goal, conservation measures that halt the alteration of habitat, particularly in diverse, older forests, will be necessary. Of course, this may come at the expense of wood production but potentially less so with forest-landscape zoning that maintains reserves, ecological forestry and spatially limited intensive management^39^.

## Methods

**Extended Data Fig. 10:**
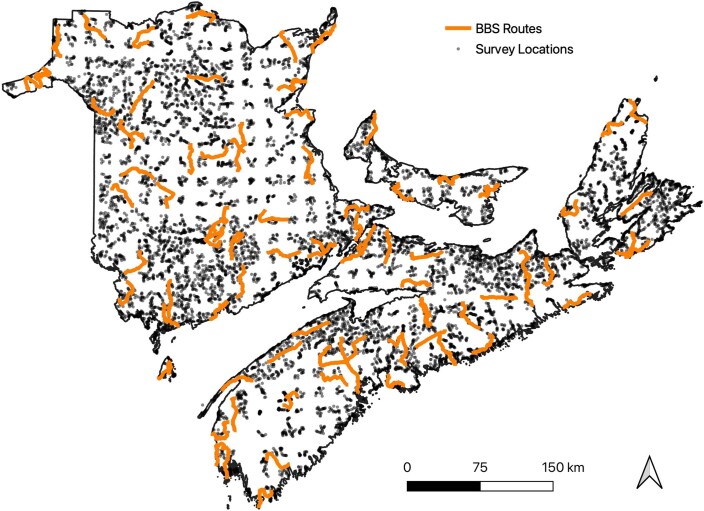
Study area and location of 12,272 Maritimes Breeding Bird Atlas (MBBA) survey locations (black dots), and Breeding Bird Survey (BBS) routes (orange lines). We used MBBA bird point counts (collected 2006–2011) to build species distribution (habitat) models, and we used long-term BBS routes (N = 90) to test whether changes in habitat in landscapes surrounding these routes successfully predicted longterm population trends in 54 species of forest birds.

We used the following overall methodology to test the hypothesis that forest degradation has resulted in bird-habitat declines. First, we used 12,272 avian point counts collected across the study region between 2006 and 2010 (Extended Data Fig. 10) with six visible Landsat reflectance bands as predictor variables to develop species distribution models for 54 bird species. We term locations with high predicted probability of occurrence ‘habitat’^17^. Second, we tested the prediction success of these models using 50% of the data not used in initial models (that is, hold-out data). Third, we back cast model predictions from SDMs to quantify habitat change from 1985 to 2020. Fourth, we tested whether habitat amount was associated with bird abundance as measured in a completely independent long-term dataset—the BBS—using both (1) a space-for-time approach (that is, whether landscapes with more habitat tend to have higher bird abundance) and (2) a temporal-change approach (that is, whether landscapes that have lost habitat in a particular year also experienced bird declines in the same year). Fifth, we tested whether direct measures of habitat degradation (that is, clear-cutting) reduced habitat. Under the forest-degradation hypothesis, we expected to see habitat for old forest-associated species decline over time and be strongly negatively associated with clear-cutting. Finally, we also estimated overall population trends for all species in our study. Again, we hypothesized that rates of decline should be greatest for those associated with old forest. Details of these five steps are provided below and in the Supplementary Methods.

### Bird point-count data

We selected 54 species of birds that were designated as forest associated by Partners in Flight and had sufficient data (*N* > 200 individual location records per species) in the Maritimes Breeding Bird Atlas (MBBA)^40^ point-count dataset to facilitate distribution modelling. Between 2006 and 2010, avian point counts were conducted at 12,272 points across three Canadian provinces: New Brunswick, Nova Scotia and Prince Edward Island (Extended Data Fig. 10). These provinces represent the core of the Acadian forest in Canada and encompass >130,000 km^2^. Point counts were conducted from May 29 to July 3, no earlier than 30 min before sunrise and no later than 5 h after sunrise. Counts were 5 min long, and species were recorded within an unlimited radius. Points were located to ensure maximum coverage of MBBA squares^40^; the coverage goal was to complete 10–15 point counts in each 10 km^2^ atlas square. Most points were randomly placed along roads, but a small proportion (8.4%; *N* = 1,034) were conducted off road. These points were placed >100 m from roads and were spaced >300 m apart.

### Remote-sensing data as predictor variables in SDMs

We followed the methods of Shirley et al.^18^ to model the distribution of species’ habitat as a function of six visible Landsat bands that we used as predictor variables in our SDMs. Using Google Earth Engine, we obtained cloud-free spectral surface reflectance from Landsat collection 1 Tier 1 from 2006 to October 2010 for building and testing SDMs. In addition, we used reflectance bands to create harmonic fitting to capture the cyclical reflectance change due to vegetation phenology and disturbance. Landsat data are collected at 30 m pixel resolution. We used the continuous change detection and classification (CCDC) algorithm^41^ in Google Earth Engine to fit each of the six Landsat spectral bands in the form of:

$$R_t = A_0 + B_0t + \mathop {\sum}\nolimits_{k = 1}^3 {\left\{ {A_k\cos \left( {\frac{{2\pi }}{T}kt} \right) + B_k\sin \left( {\frac{{2\pi }}{T}kt} \right)} \right\}}$$

where *R*_*t*_ is surface reflectance at time *t* (represented as day of year) for a spectral band, *A*_0_ is intercept, *B*_0_ is the inter-annual trend (slope) of surface reflectance, *A*_*k*_ and *B*_*k*_ are the coefficients for intra-annual spectral change and *k* is temporal frequency of harmonic components (*k* = 1, 2 and 3). *T* represents the number of days in a year (*T* = 365.25). CCDC detects where change occurs in the spectral trajectory. The advantage of this approach is that it capitalizes on (1) within-year changes in reflectance (for example, differential rates of leaf out across tree species) and (2) among-year changes in reflectance caused by disturbance and regrowth to add additional forest composition information to raw reflectance bands. The harmonic coefficients (eight coefficients) for each band (six bands) and six root-mean-squared errors from the harmonic fit were used as environmental variables in the Maxent model (54 variables; SDMs section below).

### SDMs

We used ‘Maxent’ in Google Earth Engine (equivalent of version 3.4.4) (ref. ^42^) to construct presence-only SDMs for the occurrence of 54 forest-associated species. Bird occurrence data were from the MBBA, and predictor variables constituted only the remotely sensed variables described above. Maxent uses presence-only data to predict species distributions based on maximum entropy theory. The algorithm estimates a probability distribution for species occurrence that is closest to uniform while still subject to environmental constraints (in this case, Landsat predictor variables). We generated a random sample of 10,000 pixels from the study area to serve as background samples (‘pseudo-absences’). Points sampled along roads were moved up to 180 m to the most proximate forest patch from the point-count location. The SDMs were constructed in Google Earth Engine using the linear, product and quadratic feature types provided by Maxent. A regularization multiplier was optimized by iterating the beta parameter from 0.1 to 2.0 for all 54 species separately, and the beta parameter with the highest AUC (area under the receiver operating characteristics curve) value for the model training dataset was picked to create the final Maxent model. Randomly selected model test data may not be spatially independent from data used to train SDMs thereby inflating estimates of model-prediction success. We therefore validated our models using a spatial blocking approach which separates 20% test data from 80% training data using 15 km^2^ blocks^43^ (Supplementary Fig. 4). We evaluated the performance of predictions from SDMs on validation data using AUC. The value of AUC ranges from 0 to 1. An AUC value of 0.50 indicates that the model did not perform better than random, whereas a value of 1.0 indicates perfect discrimination^44^. Note that we also tested whether SDMs successfully predicted bird abundance in an independent BBS dataset (below). Finally, we acquired Landsat images and calculated predictor variables (Remote-sensing data, above) for the 1985–2020 period and used SDMs to back cast SDM predictions for each species across the entire region in each year (for example, Fig. 3b–e). We binarized continuous SDM habitat suitability maps into habitat/non-habitat and selected species-specific cut points in the probability of occurrence that minimized false positive and negative error (Supplementary Table 1). Google Earth Engine scripts for Landsat data analysis (CCDC) and Maxent models are available at 10.6084/m9.figshare.14522322.

### BBS data, population trends and habitat loss effects

To test whether habitat change—measured using back-cast SDMs—predicted population trends, we compiled forest bird-population data from the BBS^28^^,45^between 1985 and 2019 within the boundary of the Maritime provinces of Canada (New Brunswick, Nova Scotia, Prince Edward Island), which represents the core of the Acadian forest in Canada and encompasses >130,000 km^2^. Testing for correlations between habitat and population abundance also constitutes a strong independent validation of our SDMs; if SDMs contained no information about the distribution of bird habitat, we should see little effect of modelled habitat on bird abundance. The BBS consists of a set of routes, each 40 km in length, along secondary roads surveyed annually by trained observers since 1966 (not all routes were surveyed every year). Observers stopped at 50 regularly spaced locations within each landscape and recorded the species of every bird observed during 3 min surveys. We combined data at each stop to provide the total number of individuals of each species seen during each year within a landscape. We quantified habitat change for each species in each year within 200-m diameter buffer landscapes along each of the 90 routes (that is, 40 km x 200 m areas) and used this change as the main effect in our models. We selected this spatial scale for analysis because 100 m radius is the maximum extent within which most birds can be detected using unlimited distance counts^28,46^.

We modelled trends in 54 bird populations using a modified version of the hierarchical model described by Sauer and Link^19^. The BBS data have a complex nested structure, with counts within years and within landscapes for individual species. There are several well-known limitations of these data; counts tend to be overdispersed, observers have different skill levels and can change among years and some species are more difficult to detect in an observer’s first year of surveying. The model described by Sauer and Link attempts to address these limitations while simultaneously accounting for the complex and hierarchical structure of the data. The basic form of this model is an overdispersed Poisson regression with a covariate for year, which provides inference of trends in bird abundance within each surveyed landscape. As these models control for—but do not correct—observer bias, the model provides an index of abundance rather than true abundance of birds in each landscape. Our primary modification is the use of the route as our fundamental sampling unit, which allows us to connect habitat amount on each route to BBS data.

We used several different model structures to investigate (1) population trends by species, (2) the effect of habitat amount along each route by species (which reflects a space-for-time approach to predicting effects of habitat loss on populations) and (3) the effect of habitat change on abundance changes within each route. Statistically significant effects of either (2) or (3) would constitute strong, independent validation of our habitat models and evidence that habitat affects population size. For all models, we used the survey data from 1985 to 2019 with all 90 BBS survey routes in the provinces of New Brunswick, Nova Scotia and Prince Edward Island (model parameterization details provided in Supplementary Methods).

### Reporting Summary

Further information on research design is available in the Nature Research Reporting Summary linked to this article.

## Supplementary information

Supplementary Information Supplementary information.

Reporting Summary.

Supplementary Video 1 Video showing back-cast modelled habitat change for Blackburnian warbler (*Setophaga fusca*) in the Maritime provinces of Canada.

## Data Availability

All data used in the analyses are available at 10.6084/m9.figshare.14522322. Raw BBS data are available at: https://www.pwrc.usgs.gov/BBS/RawData/. Raw data from the MBBA are available at: https://www.birdscanada.org/naturecounts/default/searchquery.jsp. Original, unprocessed Landsat images are available from Google Earth Engine: https://developers.google.com/earth-engine/datasets/catalog/landsat. Unprocessed images are too large (>2 TB each) to provide on an open access server; we provide Python code on Figshare to enable download of relevant files.

## Code availability

All Google Earth Engine, Java-script, Jags and R code used in the analyses are available at 10.6084/m9.figshare.14522322.
