# Supplementary material for: Forest degradation drives widespread avian habitat and population declines
Source: Nat Ecol Evol. 2022 Apr 28;6(6):709–19. doi: 10.1038/s41559-022-01737-8 (PMC9177422; doi:10.1038/s41559-022-01737-8)
Supplement: Supplementary file 1 — Supplementary information. [file 41559_2022_1737_MOESM1_ESM.pdf]

nature portfolio

https://doi.org/10.1038/s41559-022-01737-8

## Supplementary information

# Forest degradation drives widespread avian habitat and population declines

In the format provided by the
authors and unedited

Supplementary Materials for

# Forest degradation drives widespread avian habitat and population declines

Matthew G. Betts*, Zhiqiang Yang, Adam S. Hadley, Adam C. Smith, Josée S. Rousseau, Joseph M. Northrup, Joseph J. Nocera, Noel Gorelick, Brian D. Gerber

*Corresponding author. Email: Matthew.Betts@oregonstate.edu

This PDF file includes:

Supplementary Methods
Supplementary Figs. 1 - 6
Supplementary Tables 1 – 5
References (1-13)

Other Supplementary Materials for this manuscript include the following:

Movie S1

**Supplementary Methods**

**Breeding Bird Survey Models**

We fit all models in JAGS[1]using the 'rjags' package[2], in the statistical software R[3]. We used four Markov Chain Monte Carlo (MCMC) chains for each model with random starting values. We optimized MCMC tuning with 1000 iterations and then sampled for 24000 iterations, discarding the first 2000 as a burn-in, and then thinned by discarding every other iteration, leaving a total of 11,000 iterations per chain. We assessed convergence by calculating the Gelman-Rubin diagnostic[4] and examining trace plots of the posterior distributions of every parameter; no parameter diagnostics indicated lack of convergence.

Regional Trends

We investigated how bird counts have changed in the study period by modeling each species' annual count at route *i*, route-observer combination *j*, and year *t* ($y_{ijt}$) as,

*Equation (1)*

$$y_{ijt} \sim \text{Poisson}(\lambda_{ijt})$$
$$\log(\lambda_{ijt}) = \alpha_j + \gamma_t + \text{first.year}_{ij}\eta + \text{year}_{it}\beta_i + \epsilon_{ijt}$$
$$\alpha_j \sim \text{Normal}(\mu_\alpha, \sigma_\alpha^2)$$
$$\gamma_t \sim \text{Normal}(0, \sigma_\gamma^2)$$
$$\beta_i \sim \text{Normal}(\mu_\beta, \sigma_\beta^2)$$
$$\epsilon_{ijt} \sim \text{Normal}(0, \sigma_\epsilon^2)$$

where $\alpha_j$ are effects of unique route-observer combinations, $\gamma_t$ are year effects, $\eta$ is an effect of an observer being in their first year of conducting surveys (the variable first.year is an indicator (1 or 0) of when a route was observed for the first time by a specific observer and a zero otherwise), $\beta_i$ is the trend for route *i* (the variable year indicates the year of the survey for each route) and $\in_{ijt}$ is general dispersion beyond that accounted for by the Poisson variance. The route-specific trends ($\beta_i$) arise from a distribution with mean $\mu_\beta$ and variance $\sigma^2$ representing the regional trend for the species, while $\alpha_j$ arise from a distribution with mean μα and variance $\sigma^2$, representing the regional average route-observer effect. We used diffuse priors on our parameters as,

*Equation (2)*

$$\mu_{\beta i}, \mu_{\beta\alpha i}, \eta \sim Normal\ (0{,}10)$$

$$\sigma^2{}_\beta, \sigma_\alpha^2, \sigma_\gamma^2, \sigma_\varepsilon^2 \sim Gamma\ (0.001{,}0.001)^{\ -1}$$

Our model generally follows Sauer and Link (2011)[5], but with one important difference. Our fundamental sampling unit is the survey-route, rather than BBS strata. We use individual survey routes in order to link local habitat changes with bird counts, and to simultaneously assess overall regional population changes over time. This allows the separate estimation of the year-effects and slopes (or trends) at each route and also assumes that individual year-effects are

controlled primarily by factors operating at broader spatial scales than the individual BBS routes.

Recently, Link et al. (2020)[6] outlined how linear trend models using the entire timespan of the BBS could lead to overestimated effects. One reason for this is because of the larger amounts of missing data early on when the BBS survey began (1966). Largely though, they found that empirical results indicate that choice of model does not result in substantially different views of population change within species. Thus, supporting the use of linear trend models. Given that our dataset started in 1985 (20 years after the initiation of the BBS), we suspected that our dataset would not lead to biases in parameter estimates associated with small sample size at the beginning of the time period. Nevertheless, we used a simulation analysis to evaluate whether trend estimates were estimable at the route-level and were accurate; in other words, we tested whether simulated datasets with known population trends, and the same structure as our existing dataset tended to overestimate the mean-level trends (across routes; i.e., $\mu_\beta$).

We simulated 500 datasets for each of 54 species used in our empirical analysis. Simulated data retained the same structure, sample size, and missingness as the empirical BBS data. Simulations were based on the linear trend model at the route-level (*equations 1 & 2)*, in which all model parameters values were equivalent to the posterior mode of the empirical species estimates. However, we fixed the overall mean-level (across route) trend to either cause a 10% annual decline ($\mu_\beta$ = -0.1) or no decline ($\mu_\beta$ = 0). We evaluated whether mean-level trends were estimable and accurate by comparing posterior modes of $\mu_\beta$ from all 500 simulations for each species by the true value. We further evaluated whether the 95% highest posterior intervals of $\mu_\beta$ included the true value. We found mean-level trends were estimable and accurate (Fig. S1). Specifically, we found the posterior modes were concentrated near the true value with no bias or estimatability issues. We also found coverage of true values was close to the expected 95% (Fig. S2). We acknowledge that under certain conditions, fitting linear models to non-linear data could result in biased parameters. This could occur if population changes are steeper in recent years when sample sizes are larger. However, (1) this bias could occur in either direction (stronger negative or positive trends), and (2) given fairly consistent habitat declines over time (see Fig. 2) we have no a priori reason to expect that population trends would be amplified in more recent years of the BBS survey.

### *Population Trend – Habitat Model*

A critical assumption in back-casting species distribution models is that the relationship between habitat and bird abundance remains constant over time (i.e., temporal stationarity). To test this, we used the BBS data above to test whether (1) bird abundance along routes in each year could be predicted as a function of the SDM-predicted habitat along each route (N=90), and (2) whether annual changes in SDM-modeled habitat change (increases or decreases) along routes in each year could predict annual bird abundance changes. This second test constitutes a highly challenging test. First, many factors can drive annual fluctuations in bird abundance (e.g., weather, phenology, conditions during migration or the wintering grounds). Second, in any given year habitat change along BBS routes can be quite small for some species; this low variation in a predictor variable can preclude high statistical power to detect effects. The first test is akin to a

‘space-for-time’ approach used in most landscape-scale studies in which habitat loss is approximated by sampling landscapes across a gradient in habitat amount[7].

To conduct these tests, we followed a similar model structure as above, but instead of year, we included the amount of habitat along a route as,

*Equation (3)*

$$y_{ijt} \sim Poisson(\lambda_{ijt})$$
$$log\left(\lambda_{ijt}\right) = \alpha_j + \gamma_t + first.year_{ij}\eta + route.mean.habitat_i\theta + route.centered.habitat_{it}\beta_i + \epsilon_{ijt}$$
$$\alpha_j \sim Normal(\mu_\alpha, \sigma_\alpha^2)$$
$$\gamma_t \sim Normal(0, \sigma_\gamma^2)$$
$$\beta_i \sim Normal(\mu_\beta, \sigma_\beta^2)$$
$$\epsilon_{ijt} \sim Normal(0, \sigma_\epsilon^2)$$

where ‘route.mean.habitat’ is the mean amount of habitat available for each route across all years, scaled across all species, while the variable ‘route.centered.habitat’ is the amount of habitat in each route and year, centered within a route, and scaled across species. The parameter $\theta$ is the effect of habitat amount across routes across the full time period (interpreted as the degree to which habitat amount along routes predicts bird abundance along routes, (1) above). $\mu_\beta$ is the average effect of habitat *within* routes (interpreted as the effect of habitat change on population abundance, (2) above). Prior distributions are defined similarly as the above models. All Jags code is available at https://figshare.com/s/72d8da46d2041c984fdb.

### *Additional Species Distribution Modeling Details*

Approximately 83% of the point counts in the Maritimes Breeding Bird Atlas were conducted at the roadside (N=10,182). Existing GPS coordinates were directly on roads for these points, where no forest-associated birds occurred. We therefore jittered points where forest birds were detected so that they were in the nearest forest patch (i.e., off the road). If we had dropped all roadside counts, this would have reduced our dataset from 12,272 to 2090, a substantial reduction that would have affected our capacity to model rarer species (23 species had <100 presences in the off-road dataset). We ran Maxent models to all 54 species using only off-road data and prediction success on 50% hold-out data was not substantively different ($\bar{x}$ AUC=0.70 vs $\bar{x}$ AUC=0.69 for full dataset including on-road counts).

### *Quantifying Mature (Old)-Forest Associations*

We used Birds of the World (BOW) to establish forest successional associations for each bird species. If BOW accounts indicated that a species was associated with “old” or “mature”, or “dead wood” we counted the species as a “mature-forest associate”. Alternatively, if the account indicated that the species was associated with “open”, “bog”, or “shrub” habitat (excluding mature species nesting in shrub understories) we classified the species as a “young-forest associate”. We also used an independent bird point count dataset (from[8] to model all available species as a function of both deciduous and coniferous trees >20 cm in diameter. We used

logistic regression with a binomial error distribution to model the presence or absence of each species as function of each of these variables. If species showed positive coefficients for either deciduous or coniferous large trees, we deemed these mature-forest associates. If a species model had a negative coefficient for both of these variables, we deemed it a young-forest associate. However, if confidence intervals overlapped zero, we categorized the species as a forest-age generalist (results in Table S4). Only 30/54 species were sufficiently abundant in this dataset to yield model estimates; in all but three cases (Black-and-White Warbler, Swainson's Thrush, Hermit Thrush) these estimates concurred with the BOW accounts. In these cases of disagreement, we used the local quantitative estimates for categorization. Finally, we checked these classifications with existing habitat categories used by the New Brunswick Department of Natural Resources[9] we found no discrepancies with our final categorization.

We examined the relationship between habitat change from 1985 - 2020 and mature forest association (Fig. 4b) using linear regression with habitat change as the dependent variable. The mature-forest association has uncertainty associated with the estimates, so a simple regression using the mean mature forest association estimate would deflate the uncertainty in this relationship. Thus, we conducted this regression in a Bayesian framework, treating the true, unobserved mature forest association as a random variable. We used the mean and standard error of the estimated mature forest association to develop a prior on these values with mean equal to the estimated mean and variance equal to the squared standard error. We fit models in JAGS, running 4 chains each for 20,000 iterations, discarding the first 10,000 iterations as burn-in and assessing convergence using the Gelman-Rubin diagnostic.

### *Estimates of Loss/Gain in Bird Numbers*

To estimate the total number of birds lost or gained across the region from 1985-2020, we first calculated net habitat loss or gain for each species. We gleaned territory size of each from the Birds of the World Database[10] and multiplied: habitat change *x* territory size. We made this calculation for both the minimum territory size observed for each species, as well as the maximum. Finally, we summed these values across all 54 species to provide lower (maximum territory size) and upper (minimum territory size) bounds on the net change in bird numbers for the Maritime provinces. The equation for this calculation is:

$$\sum_{i} ((H_{1985} - H_{2020})\ x\ T)$$

Where $H$ = habitat in each time period, and $T$ = minimum, or maximum territory size for the $i$th species. Critical assumptions of these calculations are (1) that species did not change their habitat associations over time, (2) habitat was not underutilized at some point during the time series; that is, the relationship between habitat and abundance did not show systematic bias over time. We note that because SDMs contain statistical error (they are imperfect representations of 'true' habitat distributions) estimates of birds lost due to habitat changes over time are also estimated with error because non-detections of truly occupied habitats are not accounted for in SDMs.

### *Modeling Plantation Area, Mature Forest and Clearcut Disturbance over Time*

Although forest inventory data for public land and small private woodlots are freely available for New Brunswick, no such data exist for the entire study area, and are not available each year from 1985-2020. We therefore used a distribution modeling approach to predict and then backcast plantations, mature forest types, and disturbances, similar to our bird habitat SDMs.

### *Quantifying Intensive Forestry*

We used 6524 samples of known plantations >10 years since establishment as presence points then extracted harmonic coefficients as well as the synthetic spectral images corresponding to July 1 for years 1990,1995, 2000, 2005, 2010, and 2015. We only trained and tested models within areas known to have been disturbed from 1985-2010, according to our disturbance model (see ***Clearcut Disturbances*** below). We used all 6 Landsat spectral bands (blue, green, red, nir, swir1, and swir2). Plantation presence points were determined from the New Brunswick Forest Inventory[11]. We held out 50% of the data (N=3262) for model validation. Random samples of 20,000 background locations were used for Maxent modeling. Regularization optimizations were applied by comparing the AUC with varying beta from 0.1 to 2.0. The model performance was not sensitive to regularization, so the default value of 1.0 was used. The resulting model had an AUC of 0.848. We applied the Maxent predictions to all years from 1985 to 2020. A threshold (0.33) corresponding to equal sensitivity and specificity was chosen to classify the map into plantations versus non-plantations. A spatial filter of a minimum mapping unit of 11 pixels was applied to remove the scattered small patches from the final map, and we masked the final plantation map using our disturbance map (see below) to ensure that no areas that had not been clearcut were included as plantations.

### *Old Forest Types*

Old forest communities have been defined for the Acadian region by the province of New Brunswick. Stand-level tree species composition and stand structure (i.e., canopy closure, basal area metrics, density of various diameter classes of live and dead stems, woody debris) were used to define conditions representative of old forest. These definitions have been used to identify old forest using detailed forest inventory data and facilitate management objectives for the Crown forest aimed maintaining specific amounts of a variety of old-forest conditions within each ecoregion in New Brunswick. The New Brunswick old forest strategy manages for old-forest types which combine specific old forest communities into groupings expected to encompass the needs of a suite of old forest associated wildlife species (i.e., old tolerant hardwood, old hardwood, old spruce-fir, old pine, old mixedwood, other old forest).[9] Taken together, these types encompass the major old forest conditions naturally occurring in the region. We used 24,693 observations for New Brunswick Department of Natural Resources and Energy Development 'Old Forest Types', which comprise six mature forest types that are deemed to be relevant to old-forest indicator species.

For each forest type model, we established a random sample of 10,000 background (pseudo-absence) samples. We modeled each forest type separately using Maxent as with plantations above, and held out a 50% random subset of presence and background samples to use in model testing. As in the plantation model, we used harmonic coefficients from the Landsat time series to model the mature forest. Maxent features were limited to use only product, linear, and

quadratic terms. In each model, 50% of the data were held for validation. The resulting Maxent models performed well (AUC = 0.90-0.97) and were back-cast across all years from 1985 to 2020. Thresholds corresponding to equal sensitivity and specificity were chosen to classify the map into old forest communities. We then joined all six maps together into a single map of mature forest (Fig. S3), and quantified the amount of mature forest loss over the 1985-2020 period. Note that data used to train the model were from New Brunswick only, so extrapolation was necessary to model old forest in Nova Scotia and Prince Edward Island, with the chance that both remaining old forest, and old forest decline has been underestimated in these provinces.

***Clearcut Disturbances***

We created an annual medoid composite of Landsat surface reflectance from 1984 to 2020. We used the median value of mid-greenup (day 148) and mid-greendown (day 270) (from MCD12Q2.006 Land Cover Dynamics Yearly Global 500m (Friedl et al. 2019)[12]) as the starting and ending periods for the composite. We used Landtrendr (Landsat-based detection of Trends in Disturbance and Recovery) – a standard approach in mapping disturbances. Landtrendr was processed with the Normalized Burn Ratio (NBR) from the annual composite image (Kennedy et al 2018)[13] to create a time series of change segments. We used the following parameter settings in Landtrendr: (1) disturbances detected with delta-NBR less than 0.2 were masked as non-disturbed, (2) we used a minimum mapping unit of 1 ha. For further details on our method for mapping clearcuts, see Kennedy et al. (2018)[13] and Kennedy et al. (2010)[14].

***Quantifying Deforestation versus Forest Degradation***

Although there was little change in forest cover over the time period observed (a net gain in forest of 6.5% Hermosilla et al. 2022[15] we were still interested in the degree to which forest loss (i.e., deforestation) in some regions of the study area could be contributing to habitat loss, rather than forest degradation. This could be true for species whose habitat is located near cities, that still tend to be expanding in eastern Canada. We therefore quantified a new variable – "forest loss" – that represents true land conversion from forest to a non-forest land cover designation. We implemented the decision rule that if clearcut has not regenerated within 15 years of harvest (according to Hermosilla et al. 2022), it had been converted to another land-cover type (e.g., urban, agricultural, barren land). This rule is conservative in that most clearcuts in the study region tend to regenerate with either planted or other tree species within <15 years[16]. We then calculated the proportion of habitat lost for each species due to (a) deforestation, and (b) clearcutting (defined as mature forest clearcut with subsequent tree regeneration). It was not possible for us to do this calculation for the most recent 15 years of the study period because the fate of recent clearcuts is still unknown. Total amount of habitat loss due to forest loss was <2% for all 54 species (i.e., 98% of habitat loss is attributable to changes in forest structure due to harvesting, age-class truncation, and changes in forest composition, and other unmeasured variables; Table S2).

***References***

1. Plummer, M. JAGS Version 4.0. 0 user manual. (2015).

2. Plummer, M. rjags: Bayesian graphical models using MCMC. (2015).
3. R Core Team. R: A language and environment for ## statistical computing. . (2017).
4. Gelman, A. & Rubin, D. B. Inference from Iterative Simulation Using Multiple Sequences. *Statistical Science* **7**, 457–472 (1992).
5. Sauer, J. R. & Link, W. A. Analysis of the North American Breeding Bird Survey using hierarchical models. *The Auk* **128**, 87–98 (2011).
6. Link, W. A., Sauer, J. R. & Niven, D. K. Model selection for the North American Breeding Bird Survey. *Ecological Applications* **30**, e02137 (2020).
7. Trzcinski, M. K., Fahrig, L. & Merriam, G. Independent effects of forest cover and fragmentation on the distribution of forest breeding birds. *Ecological Applications* **9**, 586–593 (1999).
8. Betts, M. G., Diamond, A. W., Forbes, G. J., Villard, M.-A. & Gunn, J. S. The importance of spatial autocorrelation, extent and resolution in predicting forest bird occurrence. *Ecological Modelling* **191**, 197–224 (2006).
9. New Brunswick Department of Natural Resources. *Old Forest Community and Old-Forest Wildlife Habitat Definitions for New Brunswick.* (2012).
10. Billerman, S. M., Keeney, B. K., Rodewald, P. G. & Schulenberg T. S. *The Birds of the World*. (2020).
11. New Brunswick Department of Natural Resources and Energy Development. New Brunswick Spatial Forest Inventory. (2010).
12. Friedl, M., Gray, J. & Sulla-Menashe, D. *MCD12Q2 MODIS/Terra+Aqua Land Cover Dynamics Yearly L3 Global 500m SIN Grid V006. 2019*. (2019).
13. Kennedy, R. E. *et al.* Implementation of the LandTrendr Algorithm on Google Earth Engine. *Remote Sensing 2018, Vol. 10, Page 691* **10**, 691 (2018).
14. Kennedy, R. E., Yang, Z. & Cohen, W. B. Detecting trends in forest disturbance and recovery using yearly Landsat time series: 1. LandTrendr - Temporal segmentation algorithms. *Remote Sensing of Environment* **114**, 2897–2910 (2010).
15. Hermosilla, T., Wulder, M. A., White, J. C. & Coops, N. C. Land cover classification in an era of big and open data: Optimizing localized implementation and training data selection to improve mapping outcomes. *Remote Sensing of Environment* **268**, 112780 (2022).
16. Salmon, L., Kershaw, J. A., Taylor, A. R., Krasowski, M. & Lavigne, M. B. Exploring Factors Influencing Species Natural Regeneration Response Following Harvesting in the Acadian Forests of New Brunswick. *Open Journal of Forestry* **6**, 199–215 (2016).

Supplementary Figures and Tables

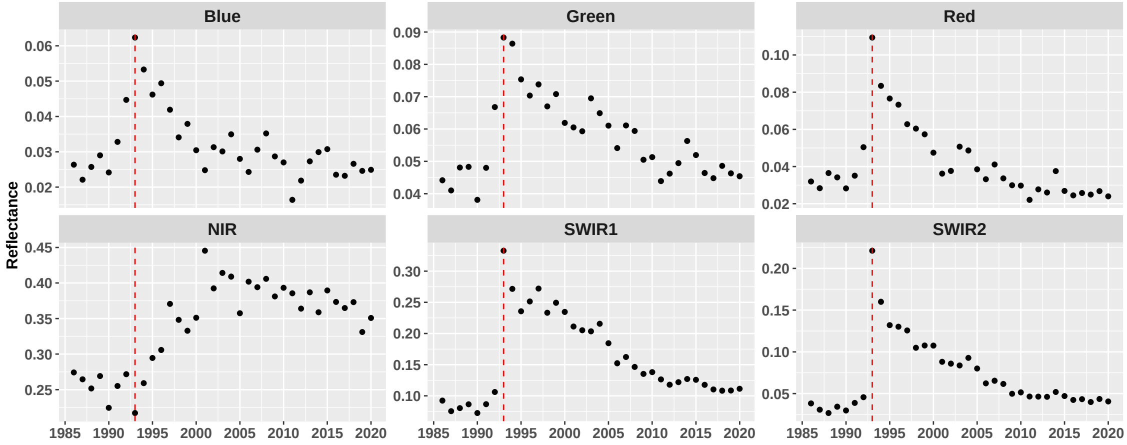


**Supplementary Fig. 1** Example of reflectance data for six Landsat bands used to model bird distributions for a single randomly selected site that was clearcut in 1993 (dashed red vertical line) and underwent regeneration over the following decades. Note the strong effect of clearcutting and regeneration on reflectance in relation to minor inter-annual reflectance change (interannual scatter), that is likely associated with climatic factors such as precipitation. This constitutes evidence that observed species-distribution modeled bird habitat declines were driven primarily by disturbance and trajectories of succession rather than factors unrelated to forest management (e.g., changes in soil moisture, vegetation phenology).

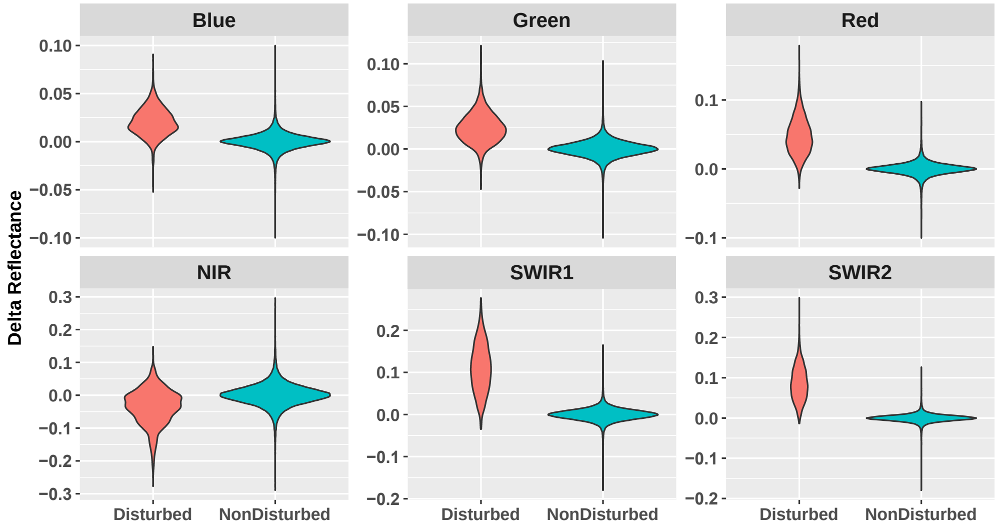


**Supplementary Fig. 2** Reflectance data for 10,000 points distributed across the entire study area showing that clearcut disturbance influences five out of six reflectance bands (Blue, Green, Red, SWIR1, SWIR2) in relation to undisturbed controls. “Delta reflectance” is the difference in reflectance from one year prior to versus one year after disturbance. Disturbed sites (red) were paired with non-disturbed sites. Although variation was present in non-disturbed sites, there was no systematic bias in reflectance over time, whereas disturbed sites differed strongly pre- versus post-disturbance.

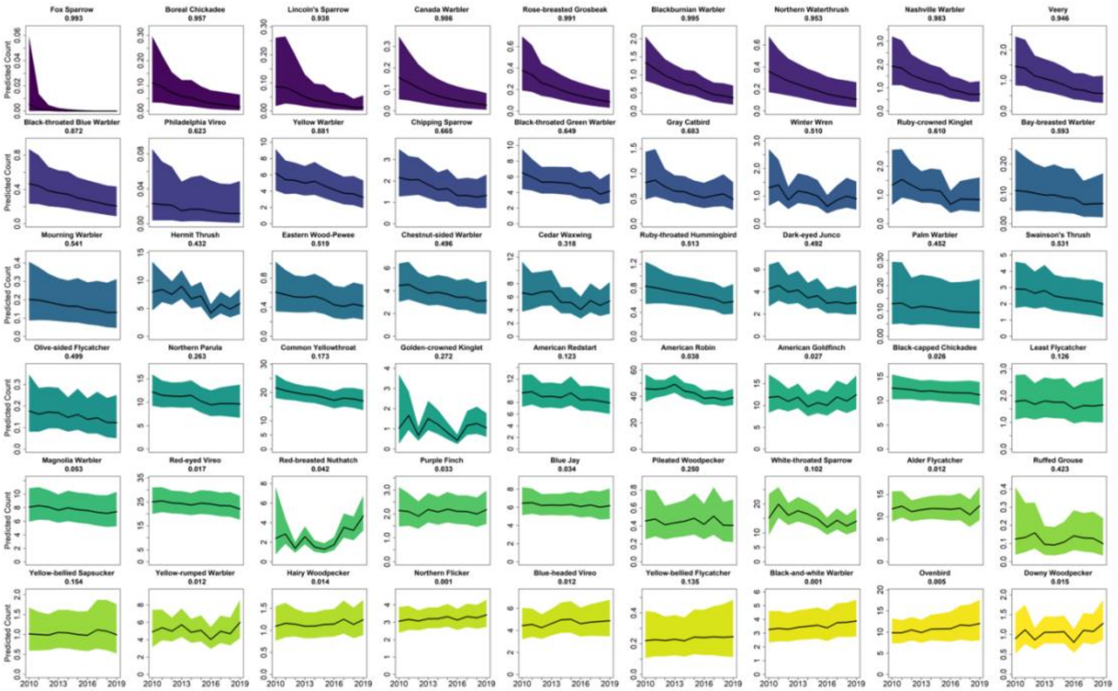


**Supplementary Fig. 3** Ten-year population trend estimates for 54 species of forest-associated birds across the Maritime Provinces of Canada. Number below species names indicate the Bayesian posterior probability that the species is declining at a rate >30% over 10 years (9 species fall into this category, 4 of which are mature-forest associated). Color-shaded areas reflect 95% credible intervals.

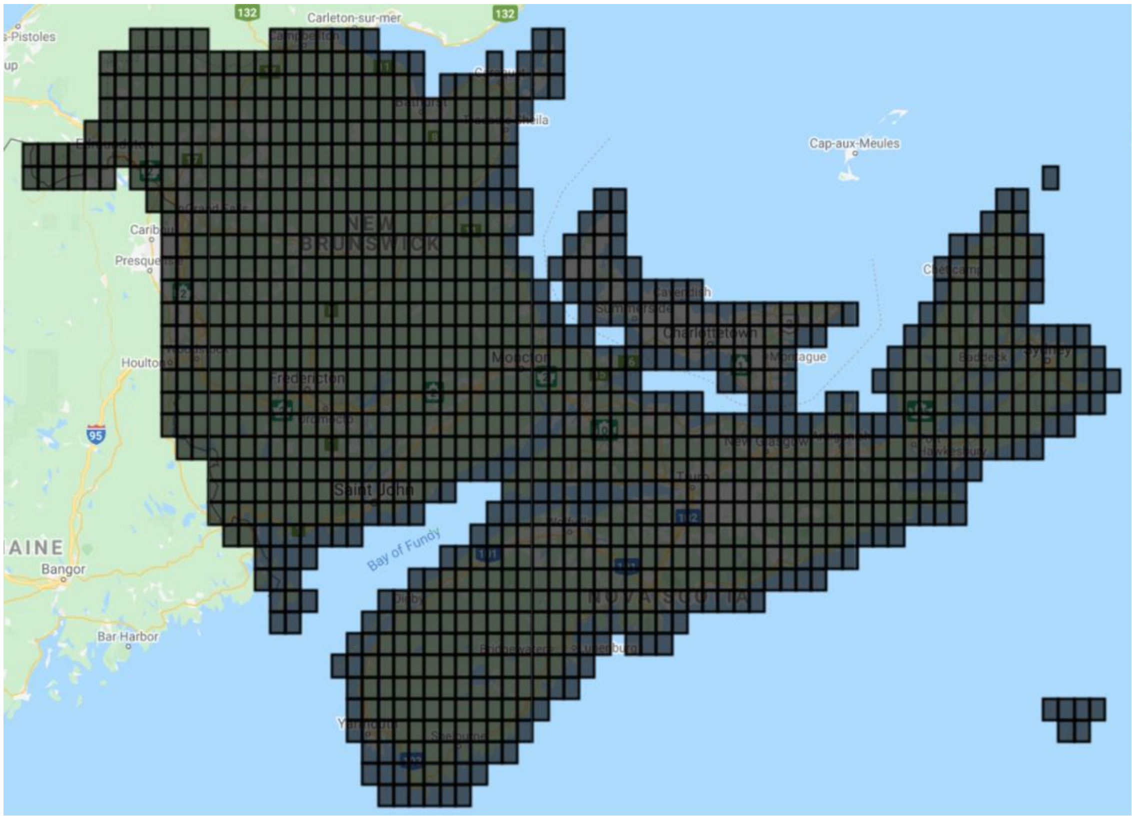


**Supplementary Fig. 4** Spatially discrete 15 km2 blocks that were randomly selected as training versus test data. Training and test data could not be contained in the same 15 km2 block.

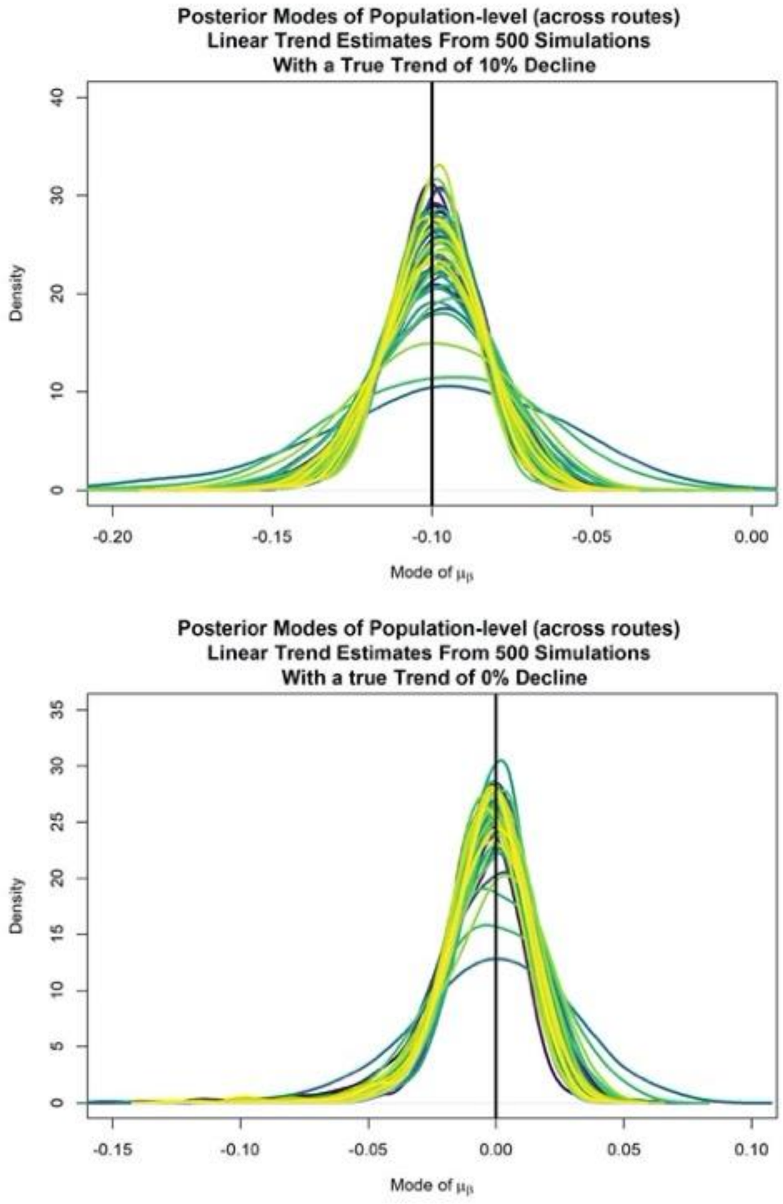


**Supplementary Fig. 5.** Plots of posterior modes of across route-level linear trend estimates from 500 simulations for each of 20 bird species. Data were simulated to have the same data and model structure as in our empirical data, but with a known slope of population change ($\mu_\beta$) to be -0.1 (10% decline) or 0 (no decline). Note that the fitted parameters generally conform to the simulated true parameters, indicating that the model and data structures do not cause systematic bias in population trend parameter estimates.

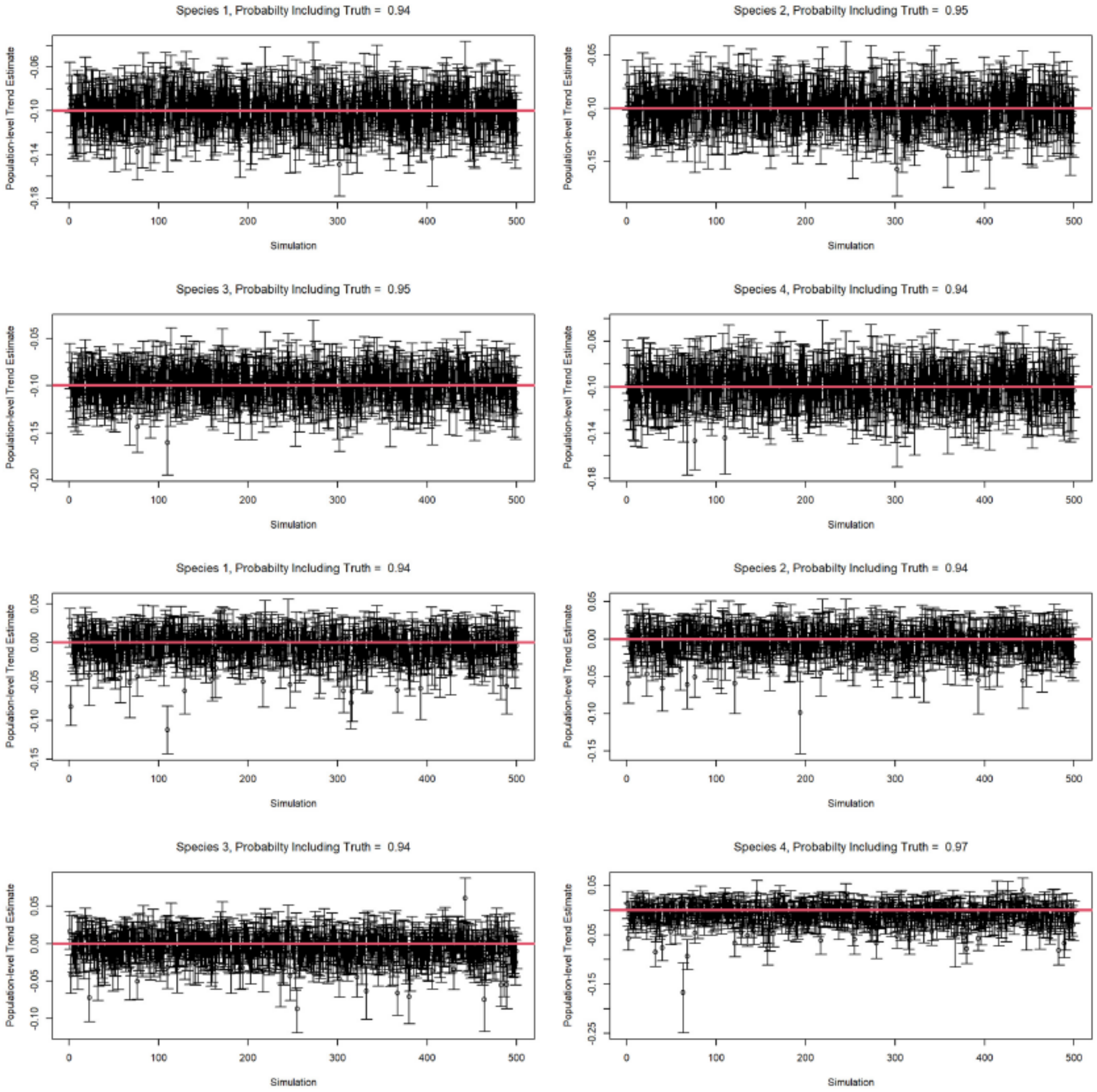


**Supplementary Fig. 6.** Four species plots of the median (open circle) and 95% highest posterior density intervals of $\mu_\beta$ for each estimate from 500 simulated datasets and two simulation scenarios. The top four plots were simulated with the true mean-level trend of -0.1 and the bottom four plots were simulated with mean-level trend of 0 (indicated by the red line). The proportion of intervals of all 500 simulations that included the true value are stated in the title.

**Supplementary Table 1**. Habitat categorizations for 54 forest bird species based on logistic regression model estimates ($\hat{\beta}$) and standard errors (SE) predicting the occurrence of each species as a function of trees >20 cm diameter (an indicator of older forest; see 'Habitat Associations'), as well as Birds of North America (BNA) species accounts. 'Age class' is the final designation of each species into regenerating (R), immature (I), and mature (M) categories. Model estimates with 'NA' are species with insufficient data.

| Species | $\hat{\beta}$ | SE | BNA | Age class | Threshold cut points in SDMs |
|---|---|---|---|---|---|
| Alder Flycatcher | NA | NA | R | R | 0.512 |
| American Goldfinch | NA | NA | R | R | 0.472 |
| American Redstart | -0.001 | 0.001 | R | I | 0.567 |
| American Robin | -0.005 | 0.001 | R | R | 0.557 |
| Bay-breasted Warbler | 0.003 | 0.001 | M | M | 0.595 |
| Black-and-white Warbler | -0.002 | 0.001 | I | I | 0.608 |
| Black-capped Chickadee | 0.001 | 0.001 | I | M | 0.544 |
| Black-throated Blue Warbler | 0.005 | 0.001 | M | M | 0.541 |
| Black-throated Green Warbler | 0.001 | 0.001 | M | M | 0.640 |
| Blackburnian Warbler | 0.003 | 0.001 | M | M | 0.618 |
| Blue Jay | NA | NA | I | M | 0.535 |
| Blue-headed Vireo | 0.002 | 0.001 | M | I | 0.650 |
| Boreal Chickadee | 0.002 | 0.001 | M | M | 0.473 |
| Canada Warbler | NA | NA | R | R | 0.622 |
| Cedar Waxwing | NA | NA | R | I | 0.497 |
| Chestnut-sided Warbler | NA | NA | R | I | 0.478 |
| Chipping Sparrow | NA | NA | R | R | 0.421 |
| Common Yellowthroat | -0.005 | 0.002 | R | R | 0.555 |
| Dark-eyed Junco | 0.002 | 0.001 | M | M | 0.65 |
| Downy Woodpecker | NA | NA | I | M | 0.523 |
| Eastern Wood-Pewee | NA | NA | I | M | 0.475 |
| Fox Sparrow | NA | NA | R | R | 0.199 |
| Golden-crowned Kinglet | 0.007 | 0.001 | M | M | 0.620 |
| Gray Catbird | NA | NA | R | R | 0.298 |
| Hairy Woodpecker | 0.002 | 0.001 | M | M | 0.596 |
| Hermit Thrush | 0.001 | 0.001 | I | M | 0.656 |
| Least Flycatcher | 0.003 | 0.001 | M | M | 0.550 |
| Lincolns Sparrow | NA | NA | R | R | 0.464 |
| Magnolia Warbler | 0.002 | 0.001 | M | I | 0.645 |
| Mourning Warbler | NA | NA | R | R | 0.444 |
| Nashville Warbler | 0.002 | 0.001 | I | R | 0.621 |
| Northern Flicker | NA | NA | R | M | 0.585 |
| Northern Parula | 0.003 | 0.001 | M | I | 0.621 |
| Northern Waterthrush | NA | NA | M | M | 0.442 |
| Olive-sided Flycatcher | NA | NA | M/R | M | 0.618 |
| Ovenbird | 0.007 | 0.001 | M | M | 0.595 |
| Palm Warbler | NA | NA | R | R | 0.605 |
| Philadelphia Vireo | NA | NA | R | I | 0.428 |
| Pileated Woodpecker | NA | NA | M | M | 0.639 |
| Purple Finch | -0.007 | 0.003 | R | M | 0.611 |
| Red-breasted Nuthatch | 0.002 | 0.001 | M | M | 0.635 |
| Red-eyed Vireo | 0.006 | 0.001 | M | M | 0.571 |
| Rose-breasted Grosbeak | NA | NA | I | I | 0.402 |
| Ruby-crowned Kinglet | -0.001 | 0.001 | R | R | 0.596 |
| Ruby-throated Hummingbird | NA | NA | R | R | 0.499 |
| Ruffed Grouse | NA | NA | I | M | 0.602 |
| Swainsons Thrush | 0.001 | 0.001 | M | M | 0.629 |
| Veery | NA | NA | I | M | 0.458 |

| | | | | | |
|---|---|---|---|---|---|
| White-throated Sparrow | -0.005 | 0.002 | R | R | 0.612 |
| Winter Wren | 0.002 | 0.001 | M | M | 0.601 |
| Yellow Warbler | NA | NA | R | R | 0.350 |
| Yellow-bellied Flycatcher | 0.002 | 0.001 | M | M | 0.622 |
| Yellow-bellied Sapsucker | 0.003 | 0.001 | I | M | 0.577 |
| Yellow-rumped Warbler | 0.002 | 0.001 | M | I | 0.640 |

**Supplementary Table 2.** Proportion of habitat lost as a function of deforestation (i.e., permanent conversion to another land-cover type).

| Species | Habitat loss (1985-2004) (ha) | Deforestation within habitat (1985-2004) (ha) | % Habitat loss due to Deforestation |
|---|---|---|---|
| Alder Flycatcher | 80974 | 839 | 1.04 |
| American Goldfinch | 138928 | 1185 | 0.85 |
| American Redstart | 127688 | 1503 | 1.18 |
| American Robin | 163702 | 1525 | 0.93 |
| Bay-breasted Warbler | 582359 | 7912 | 1.36 |
| Black-and-white Warbler | 116554 | 1301 | 1.12 |
| Black-capped Chickadee | 268097 | 2266 | 0.85 |
| Black-throated Blue Warbler | 323290 | 3227 | 1.00 |
| Black-throated Green Warbler | 382890 | 3730 | 0.97 |
| Blackburnian Warbler | 640180 | 6324 | 0.99 |
| Blue Jay | 232469 | 2069 | 0.89 |
| Blue-headed Vireo | 586262 | 6555 | 1.12 |
| Boreal Chickadee | 359872 | 4970 | 1.38 |
| Canada Warbler | 240435 | 3072 | 1.28 |
| Cedar Waxwing | 91685 | 936 | 1.02 |
| Chestnut-sided Warbler | 112250 | 1227 | 1.09 |
| Chipping Sparrow | 147420 | 1469 | 1.00 |
| Common Yellowthroat | 82346 | 894 | 1.09 |
| Dark-eyed Junco | 448041 | 5010 | 1.12 |
| Downy Woodpecker | 181563 | 1495 | 0.82 |
| Eastern Wood-Pewee | 225115 | 1976 | 0.88 |
| Fox Sparrow | 148482 | 2612 | 1.76 |
| Golden-crowned Kinglet | 778300 | 9397 | 1.21 |
| Gray Catbird | 101038 | 893 | 0.88 |
| Hairy Woodpecker | 321279 | 2923 | 0.91 |
| Hermit Thrush | 404924 | 5617 | 1.39 |
| Least Flycatcher | 179433 | 1830 | 1.02 |
| Lincolns Sparrow | 93125 | 1454 | 1.56 |
| Magnolia Warbler | 377008 | 5010 | 1.33 |
| Mourning Warbler | 107601 | 1374 | 1.28 |
| Nashville Warbler | 279299 | 4500 | 1.61 |
| Northern Flicker | 121090 | 1242 | 1.03 |
| Northern Parula | 214839 | 2199 | 1.02 |
| Northern Waterthrush | 308242 | 3875 | 1.26 |
| Olive-sided Flycatcher | 313799 | 3717 | 1.18 |

| | | | |
|---|---|---|---|
| Ovenbird | 267399 | 2559 | 0.96 |
| Palm Warbler | 118293 | 1743 | 1.47 |
| Philadelphia Vireo | 76930 | 1108 | 1.44 |
| Pileated Woodpecker | 335406 | 2898 | 0.86 |
| Purple Finch | 230337 | 2270 | 0.99 |
| Red-breasted Nuthatch | 761657 | 9145 | 1.20 |
| Red-eyed Vireo | 169751 | 1731 | 1.02 |
| Rose-breasted Grosbeak | 99306 | 1119 | 1.13 |
| Ruby-crowned Kinglet | 287897 | 3922 | 1.36 |
| Ruby-throated Hummingbird | 144531 | 1375 | 0.95 |
| Ruffed Grouse | 299767 | 2881 | 0.96 |
| Swainson’s Thrush | 466076 | 6409 | 1.38 |
| Veery | 124096 | 1322 | 1.06 |
| White-throated Sparrow | 168775 | 2468 | 1.46 |
| Winter Wren | 545377 | 6583 | 1.21 |
| Yellow Warbler | 71232 | 662 | 0.93 |
| Yellow-bellied Flycatcher | 338720 | 4941 | 1.46 |
| Yellow-bellied Sapsucker | 181196 | 1906 | 1.05 |
| Yellow-rumped Warbler | 453537 | 5463 | 1.20 |
| Total | 14420860 | 166632 | 1.16 |

**Supplementary Table 3.** Linear regression estimates for the relationship between clearcutting within 200 m of Breeding Bird Survey routes (N=90) and habitat loss in the same areas (depicted in Fig. 4c). Note that habitat loss is strongly affected by clearcutting (which is a key factor promoting forest degradation). If habitat were regenerating at the same rate as clearcut harvest, these relationships would be obscured (habitat loss due to harvest would be matched by habitat gain due to succession). Statistical tests were two-sided; no adjustments were made for cross-species multiple comparisons.

| Species | $\hat{\beta}$ | SE | $t$ | $p$ |
|---|---|---|---|---|
| Blackburnian warbler | 1.034 | 0.090 | 5.189 | $3.44 \times 10^{-19}$ |
| Bay-breasted warbler | 0.991 | 0.026 | 38.72 | $4.601 \times 10^{-57}$ |
| Red-breasted nuthatch | 0.995 | 0.069 | 14.46 | $5.67 \times 10^{-25}$ |
| Dark-eyed junco | 0.650 | 0.147 | 4.416 | $2.85 \times 10^{-5}$ |
| Golden-crowned kinglet | 1.045 | 0.053 | 19.85 | $2.89 \times 10^{-34}$ |
| Blue-headed vireo | 0.789 | 0.094 | 8.352 | $8.83 \times 10^{-13}$ |

**Supplementary Table 4.** Predicted effects of SDM-modeled habitat amount on bird abundance as measured along Breeding Bird Survey (BBS) routes. The first two columns show the range in habitat amount (in hectares) from the routes with the lowest to highest habitat amount for each species. Corresponding estimated abundances at minimum and maximum habitat along with upper 95% (UCL) and lower 95% credible intervals (LCL), and the overall effect size (maximum/minimum abundance) are provided.

| | Habitat amount (min.) | Habitat amount (max.) | Abundance at min. habitat | LCL, UCL | Abundance at max. habitat | LCL, UCL | Effect size |
|---|---|---|---|---|---|---|---|
| Alder Flycatcher | 42.44 | 771.62 | 1.71 | (1.32, 2.20) | 39.32 | (33.28, 46.52) | 23.06 |
| American Goldfinch | 12.66 | 759.37 | 1.47 | (1.15, 1.88) | 28.49 | (24.07, 33.70) | 19.34 |
| American Redstart | 66.46 | 686.10 | 5.31 | (4.27, 6.63) | 13.65 | (11.30, 16.51) | 2.57 |
| American Robin | 25.16 | 819.26 | 20.56 | (18.21, 23.28) | 77.70 | (70.75, 85.22) | 3.78 |
| Bay-breasted Warbler | 7.91 | 547.26 | 0.15 | (0.09, 0.22) | 0.74 | (0.42, 1.23) | 5.00 |
| Black-and-white Warbler | 5.57 | 782.20 | 1.32 | (0.98, 1.77) | 5.94 | (4.45, 7.89) | 4.49 |
| Blackburnian Warbler | 27.15 | 570.97 | 3.32 | (2.61, 4.22) | 10.84 | (9.37, 12.49) | 3.27 |
| Black-capped Chickadee | 17.40 | 813.47 | 0.15 | (0.10, 0.21) | 6.98 | (3.69, 13.13) | 47.79 |
| Black-throated Blue Warbler | 2.44 | 467.28 | 1.11 | (0.85, 1.43) | 7.59 | (6.07, 9.50) | 6.87 |
| Black-throated Green Warbler | 7.73 | 756.52 | 0.23 | (0.15, 0.35) | 5.53 | (2.79, 10.95) | 24.06 |
| Blue Jay | 16.90 | 791.26 | 1.77 | (1.36, 2.28) | 11.66 | (9.65, 14.08) | 6.60 |
| Blue-headed Vireo | 42.10 | 777.73 | 1.77 | (1.35, 2.29) | 10.88 | (7.66, 15.46) | 6.15 |
| Boreal Chickadee | 48.25 | 501.60 | 0.05 | (0.03, 0.08) | 0.77 | (0.44, 1.28) | 15.65 |
| Canada Warbler | 5.77 | 564.07 | 0.11 | (0.07, 0.17) | 1.59 | (0.89, 2.79) | 14.94 |
| Cedar Waxwing | 31.46 | 784.04 | 1.04 | (0.73, 1.48) | 13.16 | (10.08, 17.14) | 12.62 |
| Chestnut-sided Warbler | 7.91 | 753.57 | 0.60 | (0.43, 0.82) | 10.81 | (8.21, 14.18) | 18.03 |
| Chipping Sparrow | 58.19 | 688.11 | 0.35 | (0.26, 0.47) | 9.26 | (7.32, 11.71) | 26.28 |
| Common Yellowthroat | 16.09 | 813.45 | 3.23 | (2.69, 3.85) | 48.74 | (43.10, 55.28) | 15.09 |
| Dark-eyed Junco | 29.15 | 784.06 | 1.94 | (1.54, 2.44) | 26.00 | (19.61, 34.50) | 13.43 |
| Downy Woodpecker | 3.51 | 782.73 | 0.42 | (0.28, 0.63) | 1.37 | (0.96, 1.95) | 3.24 |
| Eastern Wood-Pewee | 15.85 | 685.20 | 0.13 | (0.09, 0.19) | 5.67 | (3.76, 8.51) | 43.28 |
| Fox Sparrow | 2.22 | 573.59 | 0.00 | (0.00, 0.00) | 4.25 | (0.46, 23.55) | 29523.87 |
| Golden-crowned Kinglet | 18.37 | 578.33 | 0.58 | (0.41, 0.82) | 2.43 | (1.57, 3.73) | 4.15 |
| Gray Catbird | 4.87 | 704.39 | 0.11 | (0.07, 0.17) | 3.74 | (2.65, 5.28) | 34.92 |
| Hairy Woodpecker | 52.61 | 834.40 | 0.93 | (0.62, 1.38) | 0.69 | (0.44, 1.08) | 0.74 |
| Hermit Thrush | 27.62 | 656.14 | 4.36 | (3.57, 5.31) | 15.68 | (12.39, 19.81) | 3.60 |
| Least Flycatcher | 32.81 | 707.21 | 0.83 | (0.59, 1.15) | 4.36 | (3.00, 6.31) | 5.28 |
| Lincolns Sparrow | 46.67 | 596.24 | 0.06 | (0.03, 0.10) | 0.37 | (0.20, 0.63) | 6.14 |
| Magnolia Warbler | 79.59 | 676.67 | 3.89 | (3.17, 4.74) | 24.07 | (19.11, 30.29) | 6.19 |
| Mourning Warbler | 23.65 | 636.52 | 0.11 | (0.06, 0.17) | 0.74 | (0.43, 1.20) | 6.96 |
| Nashville Warbler | 16.25 | 654.18 | 1.06 | (0.79, 1.39) | 3.71 | (2.69, 5.06) | 3.52 |

| | | | | | | | |
|---|---|---|---|---|---|---|---|
| Northern Flicker | 38.90 | 847.30 | 1.83 | (1.40, 2.39) | 4.14 | (3.35, 5.10) | 2.27 |
| Northern Parula | 11.43 | 748.05 | 1.88 | (1.43, 2.46) | 30.28 | (22.85, 40.13) | 16.10 |
| Northern Waterthrush | 28.61 | 602.18 | 0.14 | (0.09, 0.20) | 2.04 | (1.29, 3.15) | 14.69 |
| Olive-sided Flycatcher | 36.08 | 670.01 | 0.12 | (0.08, 0.16) | 1.49 | (0.96, 2.31) | 12.85 |
| Ovenbird | 16.41 | 684.39 | 1.10 | (0.86, 1.41) | 145.83 | (109.33, 194.74) | 132.42 |
| Palm Warbler | 1.94 | 656.19 | 0.08 | (0.05, 0.12) | 0.96 | (0.54, 1.62) | 12.36 |
| Philadelphia Vireo | 0.02 | 660.59 | 0.01 | (0.00, 0.02) | 0.11 | (0.03, 0.37) | 14.64 |
| Pileated Woodpecker | 22.87 | 787.52 | 0.26 | (0.17, 0.41) | 0.33 | (0.19, 0.54) | 1.24 |
| Purple Finch | 34.65 | 752.94 | 2.66 | (1.89, 3.75) | 2.10 | (1.62, 2.71) | 0.79 |
| Red-breasted Nuthatch | 110.59 | 564.15 | 1.06 | (0.81, 1.37) | 3.52 | (2.52, 4.89) | 3.32 |
| Red-eyed Vireo | 21.08 | 752.21 | 4.40 | (3.56, 5.40) | 45.79 | (38.27, 54.67) | 10.41 |
| Rose-breasted Grosbeak | 18.42 | 579.49 | 0.17 | (0.10, 0.27) | 1.38 | (0.89, 2.12) | 8.14 |
| Ruby-crowned Kinglet | 28.91 | 639.40 | 1.40 | (1.04, 1.86) | 5.00 | (3.59, 6.89) | 3.57 |
| Ruby-throated Hummingbird | 13.20 | 826.01 | 0.15 | (0.10, 0.24) | 2.14 | (1.35, 3.37) | 13.94 |
| Ruffed Grouse | 10.39 | 671.90 | 0.04 | (0.02, 0.07) | 0.43 | (0.20, 0.89) | 11.33 |
| Swainson’s Thrush | 1.37 | 713.54 | 2.63 | (2.07, 3.31) | 12.27 | (9.19, 16.34) | 4.67 |
| Veery | 15.10 | 613.35 | 0.32 | (0.22, 0.45) | 9.04 | (6.37, 12.69) | 28.16 |
| White-throated Sparrow | 191.91 | 650.68 | 14.82 | (12.54, 17.55) | 26.53 | (22.55, 31.25) | 1.79 |
| Winter Wren | 8.87 | 716.60 | 0.89 | (0.67, 1.17) | 6.80 | (4.75, 9.64) | 7.62 |
| Yellow Warbler | 5.71 | 710.81 | 1.15 | (0.93, 1.43) | 18.68 | (15.66, 22.17) | 16.19 |
| Yellow-bellied Flycatcher | 3.39 | 526.08 | 0.26 | (0.19, 0.36) | 2.06 | (1.38, 3.00) | 7.84 |
| Yellow-bellied Sapsucker | 0.58 | 622.34 | 0.22 | (0.15, 0.31) | 7.12 | (4.79, 10.47) | 32.50 |
| Yellow-rumped Warbler | 13.69 | 769.58 | 2.53 | (2.05, 3.11) | 15.43 | (11.96, 19.90) | 6.09 |

Supplementary Table 5. Hectares of habitat lost (‘Loss’, 1985-2020) minimum (Min) and maximum (Max) reported territory sizes and estimated minimum and maximum number of birds lost (Min x Loss, Max x Loss) for 54 species of forest birds.

| Species | Code | Loss (ha) | Min (ha) | Max (ha) | Min. lost | Max. lost |
|---|---|---|---|---|---|---|
| Alder Flycatcher | ALFL | 302,278 | 0.20 | 3.00 | 100,759 | 1,511,390 |
| American Goldfinch | AMGO | -496,899 | NA | NA | NA | NA |
| American Redstart | AMRE | 143,888 | 0.39 | 1.00 | 143,888 | 368,944 |
| American Robin | AMRO | -461,831 | 0.11 | 0.84 | -549,799 | -4,198,462 |
| Bay-breasted Warbler | BBWA | -1,384,012 | 1.50 | 1.50 | -922,675 | -922,675 |
| Black-and-white Warbler | BAWW | 217,677 | 1.93 | 3.57 | 60,950 | 112,870 |
| Black-capped Chickadee | BCCH | -686,409 | 1.50 | 5.30 | -129,511 | -457,606 |
| Black-throated Blue Warbler | BTBW | -382,489 | 1.00 | 4.00 | -95,622 | -382,489 |
| Black-throated Green Warbler | BTNW | -1,094,419 | 0.25 | 0.90 | -1,216,021 | -4,377,674 |
| Blackburnian Warbler | BLBW | -1,908,373 | 0.40 | 1.10 | -1,734,885 | -4,770,933 |
| Blue Jay | BLJA | -557,514 | NA | NA | NA | NA |
| Blue-headed Vireo | BHVI | -1,838,962 | 3.00 | 3.00 | -612,987 | -612,987 |
| Boreal Chickadee | BOCH | -895,580 | 5.00 | 5.00 | -179,116 | -179,116 |
| Canada Warbler | CAWA | 118,590 | 0.24 | 0.80 | 148,238 | 494,127 |
| Cedar Waxwing | CEWA | 209,893 | NA | NA | NA | NA |
| Chestnut-sided Warbler | CSWA | 315,671 | 0.40 | 1.10 | 286,973 | 789,176 |
| Chipping Sparrow | CHSP | -137,187 | 0.20 | 1.00 | -137,187 | -685,937 |
| Common Yellowthroat | COYE | 370,919 | 0.20 | 2.20 | 168,600 | 1,854,597 |
| Dark-eyed Junco | DEJU | -2,129,036 | 1.31 | 1.96 | -1,086,243 | -1,625,218 |
| Downy Woodpecker | DOWO | -113,816 | 4.40 | 5.50 | -20,694 | -25,867 |
| Eastern Wood-Pewee | EAWP | -565,774 | 2.20 | 7.70 | -73,477 | -257,170 |
| Fox Sparrow | FOSP | 1,993 | 0.25 | 1.00 | 1,993 | 7,971 |
| Golden-crowned Kinglet | GCKI | -2,340,504 | 0.34 | 1.60 | -1,462,815 | -6,965,785 |
| Gray Catbird | GRCA | -96,834 | 0.32 | 0.41 | -236,180 | -302,606 |
| Hairy Woodpecker | HAWO | -1,164,827 | 0.65 | 1.50 | -776,551 | -1,792,041 |
| Hermit Thrush | HETH | -815,713 | 0.72 | 3.34 | -244,225 | -1,129,796 |
| Least Flycatcher | LEFL | -192,566 | 0.11 | 0.43 | -447,829 | -1,750,604 |
| Lincolns Sparrow | LISP | 497,618 | 1.43 | 2.00 | 248,809 | 348,333 |
| Magnolia Warbler | MAWA | -1,267,549 | 0.51 | 1.05 | -1,207,190 | -2,485,391 |
| Mourning Warbler | MOWA | 25,130 | 0.50 | 0.65 | 38,662 | 50,260 |
| Nashville Warbler | NAWA | -26,507 | 1.10 | 1.10 | -24,098 | -24,098 |
| Northern Flicker | NOFL | 93,575 | 50.00 | 111.00 | 843 | 1,872 |
| Northern Parula | NOPA | -388,443 | 0.08 | 0.65 | -597,605 | -4,855,539 |
| Northern Waterthrush | NOWA | -886,875 | 0.80 | 1.50 | -591,250 | -1,108,594 |
| Olive-sided Flycatcher | OSFL | -814,650 | 10.00 | 40.00 | -20,366 | -81,465 |
| Ovenbird | OVEN | -646,331 | 0.48 | 0.83 | -781,536 | -1,349,333 |
| Palm Warbler | PIWA | 539,477 | 0.70 | 13.70 | 39,378 | 770,681 |
| Philadelphia Vireo | PHVI | 251,573 | 0.50 | 4.00 | 62,893 | 503,146 |
| Pileated Woodpecker | PIWO | -619,808 | NA | NA | NA | NA |
| Purple Finch | PUFI | -439,157 | 62.50 | 83.33 | -5,270 | -7,027 |
| Red-breasted Nuthatch | RBNU | -1,960,231 | 0.20 | 10.00 | -196,023 | -9,801,154 |

| | | | | | | |
|---|---|---|---|---|---|---|
| Red-eyed Vireo | REVI | -432,886 | 0.86 | 3.71 | -116,681 | -503,355 |
| Rose-breasted Grosbeak | RBGR | 14,645 | 0.34 | 1.30 | 11,265 | 43,072 |
| Ruby-crowned Kinglet | RUKI | -1,023,415 | 1.10 | 6.00 | -170,569 | -930,378 |
| Ruby-throated Hummingbird | RTHU | 88,815 | NA | NA | NA | NA |
| Species | | Loss | Min (ha) | Max (ha) | Min. Lost | Max. lost |
| Ruffed Grouse | RUGR | -598,188 | 2.10 | 2.30 | -260,082 | -284,851 |
| Swainsons Thrush | SWTH | -952,748 | 2.10 | 2.10 | -453,689 | -453,689 |
| Veery | VEER | -43,794 | 0.10 | 2.00 | -21,897 | -437,937 |
| White-throated Sparrow | WTSP | 42,618 | 0.99 | 3.26 | 13,073 | 43,049 |
| Winter Wren | WIWR | -1,240,884 | 1.90 | 2.10 | -590,897 | -653,097 |
| Yellow Warbler | YEWA | -177,218 | 0.20 | 0.78 | -227,203 | -886,092 |
| Yellow-bellied Flycatcher | YBFL | -1,025,003 | 0.75 | 1.00 | -1,025,003 | -1,366,671 |
| Yellow-bellied Sapsucker | YBSA | -175,882 | 0.81 | 3.10 | -56,736 | -217,139 |
| Yellow-rumped Warbler | YRWA | -1,467,292 | 0.45 | 0.80 | -1,834,115 | -3,260,648 |
| Males lost | | -28,215,247 | | | -16,779,704 | -52,243,938 |
| Individuals lost | | | | | -33,559,408 | -104,487,876 |

Movie S1.

Animation showing species distribution model using Landsat TM bands for blackburnian warbler (*Setophaga fusca*) in each year of our study (1985-2020) for the Maritime Provinces of Canada. Habitat for this mature-forest associated species (delineated in blue) declined 33% over the period observed. This habitat change was driven primarily by clearcutting without sufficient habitat regeneration (due to tree species composition changes and age-class truncation), and was a strong predictor of population declines in this species.
