# Supplementary material for: Forest degradation drives widespread avian habitat and population declines
Source: Nat Ecol Evol. 2022 Apr 28;6(6):709–19. doi: 10.1038/s41559-022-01737-8 (PMC9177422; doi:10.1038/s41559-022-01737-8)
Supplement: Supplementary file 2 — Reporting Summary. [file 41559_2022_1737_MOESM2_ESM.pdf]

nature portfolio

Corresponding author(s): Matthew G Betts

Last updated by author(s): Mar 11, 2022

Reporting Summary

Nature Portfolio wishes to improve the reproducibility of the work that we publish. This form provides structure for consistency and transparency in reporting. For further information on Nature Portfolio policies, see our Editorial Policies and the Editorial Policy Checklist.

Statistics

For all statistical analyses, confirm that the following items are present in the figure legend, table legend, main text, or Methods section.

n/a	Confirmed	
☐	☒	The exact sample size (*n*) for each experimental group/condition, given as a discrete number and unit of measurement
☐	☒	A statement on whether measurements were taken from distinct samples or whether the same sample was measured repeatedly
☐	☒	The statistical test(s) used AND whether they are one- or two-sided<br>*Only common tests should be described solely by name; describe more complex techniques in the Methods section.*
☐	☒	A description of all covariates tested
☐	☒	A description of any assumptions or corrections, such as tests of normality and adjustment for multiple comparisons
☐	☒	A full description of the statistical parameters including central tendency (e.g. means) or other basic estimates (e.g. regression coefficient) AND variation (e.g. standard deviation) or associated estimates of uncertainty (e.g. confidence intervals)
☐	☒	For null hypothesis testing, the test statistic (e.g. F, t, r) with confidence intervals, effect sizes, degrees of freedom and P value noted<br>*Give P values as exact values whenever suitable.*
☐	☒	For Bayesian analysis, information on the choice of priors and Markov chain Monte Carlo settings
☐	☒	For hierarchical and complex designs, identification of the appropriate level for tests and full reporting of outcomes
☐	☒	Estimates of effect sizes (e.g. Cohen's *d*, Pearson's *r*), indicating how they were calculated

Our web collection on statistics for biologists contains articles on many of the points above.

Software and code

Policy information about availability of computer code

Data collection	All data used in the analyses are available at https://doi.org/10.6084/m9.figshare.14522322. Raw Breeding Bird Survey data are available at: https://www.pwrc.usgs.gov/BBS/RawData/. Raw data from the Maritimes Breeding Bird Atlas are available at: https://www.birdscanada.org/naturecounts/default/searchquery.jsp. Original, unprocessed Landsat Images are available from Google Earth Engine: https://developers.google.com/earth-engine/datasets/catalog/landsat Unprocessed images are too large (>2 TB each) to provide on an open access server; we provide Python code on Figshare to enable download of relevant files.
Data analysis	All Google Earth Engine, Java-script, Jags, and R code used in the analyses are available at https://doi.org/10.6084/m9.figshare.14522322.

For manuscripts utilizing custom algorithms or software that are central to the research but not yet described in published literature, software must be made available to editors and reviewers. We strongly encourage code deposition in a community repository (e.g. GitHub). See the Nature Portfolio guidelines for submitting code & software for further information.

Data

Policy information about availability of data

All manuscripts must include a data availability statement. This statement should provide the following information, where applicable:

- Accession codes, unique identifiers, or web links for publicly available datasets
- A description of any restrictions on data availability
- For clinical datasets or third party data, please ensure that the statement adheres to our policy

All Google Earth Engine, Java-script, Jags, and R code used in the analyses are available at https://doi.org/10.6084/m9.figshare.14522322.

Field-specific reporting

Please select the one below that is the best fit for your research. If you are not sure, read the appropriate sections before making your selection.

☐ Life sciences ☐ Behavioural & social sciences ☒ Ecological, evolutionary & environmental sciences

For a reference copy of the document with all sections, see nature.com/documents/nr-reporting-summary-flat.pdf

Ecological, evolutionary & environmental sciences study design

All studies must disclose on these points even when the disclosure is negative.

Study description	We used 12,272 avian point counts collected across the study region between 2006 and 2010 (Extended Data Fig. 10) with six visible Landsat reflectance bends as predictor variables to develop species distribution models for 54 bird species. To test whether habitat change, measured using back-cast SDMs, predicted population trends we compiled forest bird population data from the Canadian Breeding Bird Survey between 1985-2019 within the boundary of the Maritime Provinces of Canada (New Brunswick, Nova Scotia, Prince Edward Island), which represents the core of the Acadian Forest in Canada and encompasses >130,000 km2
Research sample	We used 12,272 avian point counts from the Maritimes Breeding Bird Atlas (MBBA) to build SDMs<br>We used Breeding Bird Survey (BBS) Data (N=90 routes) to test the effect of habitat change on bird populations
Sampling strategy	This study used existing data, so analysis conformed to previously established sample sizes and spatial distribution of samples available in the Maritimes Breeding Bird Atlas and the Canadian Breeding Bird Survey
Data collection	The BBS consists of a set of routes, each 40 km in length, along secondary roads surveyed annually by trained observers since 1966 (not all routes were surveyed every year). Observers stopped at 50 regularly spaced locations within each landscape and recorded the species of every bird observed during 3-minute surveys. Maritimes Breeding Bird Atlas (MBBA)40 point count dataset to facilitate distribution modeling. Between 2006 and 2010, avian point counts were conducted at 12,272 points across three Canadian provinces: New Brunswick, Nova Scotia, and Prince Edward Island (Extended Data Fig. 10). These provinces represent the core of the Acadian Forest in Canada and encompass >130,000 km2. Point counts were conducted from May 29 to July 3, no earlier than 30 minutes before sunrise and no later than 5 hours after sunrise. Counts were 5-minutes long, and species were recorded within an unlimited radius. Points were located to ensure maximum coverage of Breeding Bird Atlas squares40; the coverage goal was to complete 10-15 point counts in each 10 km2 atlas square. Most points were randomly placed along roads, but a small proportion (8.4%; N=1034) were conducted off-road. These points were placed >100 m from roads and were spaced >300 m apart.
Timing and spatial scale	Breeding Bird Survey: Distributed across the entire study region (Maritime Provinces) at 90 BBS routes over a period from 1985-2019<br>Maritimes Breeding Bird Atlas: Distributed across the entire study area at 12,272 points, collected from 2006-2010
Data exclusions	Not applicable
Reproducibility	We tested species distribution models on independent data (the Breeding Bird Survey data) to ensure that models performed well.
Randomization	As noted above, we used existing data, so no random selection was possible. Initially, BBS and MBBA locations were selected as part of a spatially stratified sample across the Maritime Provinces.
Blinding	Observers of empirical data were blind to the study hypotheses (because they were not aware of these objectives at the time of data collection).

Did the study involve field work? ☐ Yes ☐ No

Field work, collection and transport

Field conditions	Not applicable. We did not collect field data but used existing data.
Location	Maritime Provinces of Canada (Nova Scotia, New Brunswick, Prince Edward Island)
Access & import/export	Not applicable (we used existing data)
Disturbance	Not applicable (we did not engage in fieldwork)

Reporting for specific materials, systems and methods

We require information from authors about some types of materials, experimental systems and methods used in many studies. Here, indicate whether each material, system or method listed is relevant to your study. If you are not sure if a list item applies to your research, read the appropriate section before selecting a response.

Materials & experimental systems

n/a	Involved in the study
☒	☐ Antibodies
☒	☐ Eukaryotic cell lines
☒	☐ Palaeontology and archaeology
☒	☐ Animals and other organisms
☒	☐ Human research participants
☒	☐ Clinical data
☒	☐ Dual use research of concern

Methods

n/a	Involved in the study
☒	☐ ChIP-seq
☒	☐ Flow cytometry
☒	☐ MRI-based neuroimaging
